# Deception and Manipulation: The Arms of *Leishmania*, a Successful Parasite

**DOI:** 10.3389/fimmu.2014.00480

**Published:** 2014-10-20

**Authors:** Pedro Cecílio, Begoña Pérez-Cabezas, Nuno Santarém, Joana Maciel, Vasco Rodrigues, Anabela Cordeiro da Silva

**Affiliations:** ^1^Parasite Disease Group, Institute for Molecular and Cell Biology (IBMC), University of Porto, Porto, Portugal; ^2^Department of Biological Sciences, Faculty of Pharmacy, University of Porto, Porto, Portugal

**Keywords:** *Leishmania*, immunomodulation, apoptosis, innate immunity, acquired immunity

## Abstract

*Leishmania* spp. are intracellular parasitic protozoa responsible for a group of neglected tropical diseases, endemic in 98 countries around the world, called leishmaniasis. These parasites have a complex digenetic life cycle requiring a susceptible vertebrate host and a permissive insect vector, which allow their transmission. The clinical manifestations associated with leishmaniasis depend on complex interactions between the parasite and the host immune system. Consequently, leishmaniasis can be manifested as a self-healing cutaneous affliction or a visceral pathology, being the last one fatal in 85–90% of untreated cases. As a result of a long host–parasite co-evolutionary process, *Leishmania* spp. developed different immunomodulatory strategies that are essential for the establishment of infection. Only through deception and manipulation of the immune system, *Leishmania* spp. can complete its life cycle and survive. The understanding of the mechanisms associated with immune evasion and disease progression is essential for the development of novel therapies and vaccine approaches. Here, we revise how the parasite manipulates cell death and immune responses to survive and thrive in the shadow of the immune system.

## Introduction

Parasitism is defined as a “non-mutual symbiotic relationship between species, where one species, the parasite, benefits at the expense of the other, the host,” Such relationship occurs during leishmaniasis, where the protozoan *Leishmania* spp. takes advantage of its mammalian host in order to survive and thrive.

*Leishmania* is a genus of trypanosomatid protozoa that combines over 30 species, of which 11 have significant medical and veterinary importance (1). These parasites have a complex digenetic life cycle, with some particularities, requiring a vertebrate host and an insect vector. The alimentary tract of female *Phlebotomus* spp. and *Lutzomyia* spp. sandflies is colonized by the extracellular form of the parasite, the flagellated, and motile promastigote. Within the insect midgut, *Leishmania* undergoes several developmental changes that culminate in the infectious developmental form of the parasite: the metacyclic promastigote. During the insect blood feeding, the parasite infectious forms are released into the mammal host dermis and quickly uptaken by mono and polymorphonuclear (PMN) cells. Ultimately, in the phagolysosome of macrophages, promastigotes will differentiate into the non-motile amastigote form and multiply. The cycle is completed when the sandfly takes another blood meal, recovering free amastigotes or infected cells (1–3).

Leishmaniasis is endemic in 98 countries, 72 of which are developing nations and 13 correspond to the least developed ones, being considered by the World Health Organization as a Neglected Tropical Disease (4, 5). Over 350 million people reside in areas with active parasite transmission (6). Annually, an estimated 1.5–2 million develop symptomatic disease, and approximately 50,000 die, mostly children (4, 7). Climate changes and population mobility can contribute to the increase of the vector activity and, consequently of the disease incidence (8, 9). The infection caused by *Leishmania* spp. can lead to different clinical manifestations depending on complex interactions between the parasite and the host immune response. The disease is normally divided into three main categories: cutaneous, mucocutaneous, and visceral. Cutaneous leishmaniasis is the most extensively studied form of the disease, usually appearing as a self-healing skin ulcer or dermal granuloma that may need several months or years to heal (10). In some cases, these ulcers can become chronic (11). While most *Leishmania* species cause lesions confined to small areas of the skin, a few, such as *L. braziliensis*, cause diffuse lesions that may even spread to mucosal tissues leading to the mucocutaneous form of the disease (12). Finally, visceral leishmaniasis, the most severe leishmaniasis form, is caused by *Leishmania donovani* and *Leishmania infantum*. It is characterized by fever, cachexia, hepatosplenomegaly and hypergamaglobulinemia and, when untreated, can be fatal (13). In endemic countries, *Leishmania* has gained prominence as an opportunistic pathogen in HIV positive and other immunocompromised patients (8, 14). Leishmaniasis is also a major veterinary concern, as dogs are the main reservoir for the parasite in South America and southwestern Europe (15).

There is no human vaccine available at the moment. Nonetheless, prevention of infection through vaccination seems to be a viable option, since in endemic areas the majority of infected persons do not develop clinical symptoms and previous infection leads to robust immunity against the parasite (16). In the absence of vaccines, control of the disease relies on prophylaxis and treatment, reviewed elsewhere (17, 18). Treatment options are limited, present significant toxicity and require, with the exception of oral miltefosine, administration in ambulatory conditions (18). Drug resistance is also a growing limitation of some anti-leishmanial therapies (19). Therefore, it is essential to develop novel treatment options and vaccine strategies. Such goal has its cornerstone on the solid knowledge of the details of parasite infection. For this, different strategies that *Leishmania* uses to manipulate the immune system to establish infection will be revised here.

## Playing with Death Toward the Establishment and Maintenance of Infection

Apoptosis, or programed cell death, is a physiological and essential process for the maintenance of general cellular homeostasis. In immunology, this mechanism is indispensable for elimination of autoreactive immune cells (20, 21) and control of the proliferative response (22, 23). Programed cell death also plays a key role in the resolution of infections produced by intracellular pathogens (24). However, and as a result of the continuous host-microbe co-evolutionary process, *Leishmania* developed strategies for using apoptosis to its own benefit.

### Dead parasites are essential for the survival of free promastigotes

**Figure 1:**
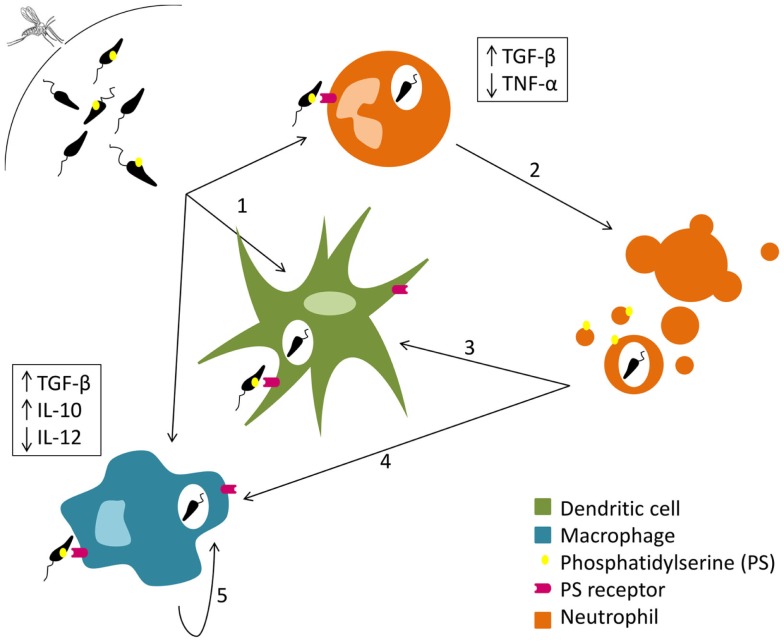
**Silent entry of *Leishmania* into the host cells**. Live and dead parasites are engulfed by phagocytes. The recognition of the externalized phosphatidylserine present on the cellular membrane of dead parasites induces TGF-β secretion and TNF-α downregulation (1). Neutrophil apoptosis is delayed by *Leishmania* (2). Both dendritic cells (3) and macrophages (4) remove neutrophil apoptotic bodies carrying *Leishmania* promastigotes and secrete TGF-β and IL-10. Macrophages (5) can also phagocyte parasites extruded within other macrophage membrane blebs, which in turn promotes the secretion of IL-10.

Parasite cell death, reviewed elsewhere (25–27), seems to be very relevant for the deception of the initial immune response. Some authors described that the presence of apoptotic parasites is essential for successful infection of mice susceptible to cutaneous leishmaniasis. Indeed BALB/c mice did not develop disease after intradermal infection with purified virulent non-apoptotic parasites (28, 29). The need for dead parasites in the infective inoculum is related with the exposure of phosphatidylserine (PS) in the outer leaflet of the parasite cytoplasmic membrane. The exposure of this phospholipid enables a silent invasion, inducing the production of anti-inflammatory cytokines such as TGF-β (30, 31). In fact, a recent study shows that the administration of a PS-targeting antibody after C57Bl/6 mice intradermal infection with *L. amazonensis* promastigotes renders the animals more resistant to the infection (32). Thereby, and as represented in Figure 1, the inoculation of equal proportions of dead and live parasites in the mammalian host may allow the silent entry of *Leishmania* into the first cells recruited to the inoculation site (28, 33).

### Modulating apoptosis of neutrophils at the inoculation site

It is accepted that macrophages are the cells predominantly infected in leishmaniasis. However, they are neither the first nor the only to be recruited to the site of inoculation. Several evidences support the early recruitment of neutrophils to the inoculation site. Two hours after natural infection of C57Bl/6 mice with *L. major*, neutrophils are predominantly found (34). Such granulocyte infiltration was also seen upon intradermal infection of either BALB/c or C57Bl/6 mice with *L. infantum* and *L. major*, respectively (35, 36), as well as after subcutaneous infection with *L. amazonensis* or *L. major* promastigotes (37–39). Furthermore in a murine air pouch model, *L. major*, and to a lower extent *L. donovani*, predominantly induced the recruitment of neutrophils 6 h after infection (40, 41). Interestingly, the air pouch system revealed that *L. major* derived extracellular vesicles induced the same type of cellular recruitment as parasites (40). These studies preceded the description of Wilson et al. who saw neutrophils infiltration 1 h after intradermal inoculation of *L. donovani* promastigotes in hamsters (42). Although the role of neutrophils during infection is not consensual, several evidences support the capacity of *Leishmania* to modulate their life span. Traditionally, neutrophils show a relatively short life span (43), but *Leishmania* can successfully delay their programed cell death for up than 24 h, potentially benefiting from the protection of a safe intracellular niche (44). However, other studies show induction of neutrophil apoptosis after parasite intake (35). These contradictions may be due to differences in the genetic background of the animal model used (BALB/c versus C57Bl/6), as well as in the parasite inoculation route (45). The delay in the natural apoptotic process of infected neutrophils was related to an inhibition of the pro-caspase-3 processing (44), and the consequent diminishment of caspase 3, a well-known apoptosis executer in neutrophils (46). Moreover, a recent publication clarified the mechanisms by which *L. major* contributes for the neutrophil apoptosis inhibition, showing that the key event is the activation of the extracellular signal-regulated kinases (ERK1/2) survival pathway (47). Sarkar and colleagues showed that the parasite upregulates ERK1/2 phosphorylation, leading to the delay of neutrophil apoptosis (48). Also, this work unveiled additional players of the apoptotic machinery responsible for neutrophil life span enhancement. Among these the anti-apoptotic proteins, Bfl-1 and Bcl-2 were upregulated, preventing the release of cytochrome *c* from the mitochondria and the downstream activation of caspases. Additionally, processing of the pro-apoptotic Bid was inhibited and the Fas expression reduced, preventing apoptosis triggering (48). This delay of neutrophil death may be essential for the arrival of a sufficient number of antigen-presenting cells (APCs), namely macrophages, and dendritic cells (DCs), to the inoculation site.

### “Trojan horse” strategy

After being infected, dying neutrophils secrete different chemotactic factors for macrophages (49, 50); cells that then remove apoptotic neutrophils by phagocytosis and secrete the anti-inflammatory cytokine TGF-β (50). High amounts of IL-10 and low amounts of interleukin (IL)-12 may also contribute for the silent entry of *L. major* into macrophages (51) as shown in Figure 1. The parasite can, therefore, arrive to its primary host cell unnoticed and proceed with the infection process, using the so-called “Trojan horse” strategy (52). TGF-β seems to be essential for the establishment of infection not only by *L. major* but also by *L. amazonensis*, although conclusions about the exploitation of the “Trojan horse” strategy in this case cannot be withdrawn (50, 53). DCs have also been related with this tactic. Ribeiro-Gomes et al. recently described in a mouse model of intradermal infection with *L. major* that skin resident DCs uptake apoptotic infected neutrophils and, as a consequence, the activation of *Leishmania*-specific CD4^+^ T cells is prevented somehow (35). Other authors suggested that free parasites silently enter into host cells taking advantage of nearby neutrophil apoptotic bodies with exposed PS (54).

### Buying time by prolonging the life of macrophages

When promastigotes reach macrophages, its definitive cellular host, a new step of the infective process begins with their differentiation into amastigotes. Therefore, inhibition of apoptosis may be once more essential for *Leishmania* to protect its niche, enabling the differentiation into the amastigote form that is fully adapted to the phagolysosome. Extensive data exists concerning the capacity of the parasite to increase the life span of infected macrophages. The first description was made by Moore and Matlashewski, who reported that *L. donovani* infection of murine bone marrow-derived macrophages (BMM) represses macrophage apoptosis through a mechanism dependent on the secretion of TNF-α (55). Since then, numerous studies addressed this issue, unveiling some intracellular mechanisms that could explain the death delay. External ATP is known to trigger death in macrophages when injured or stressed, by its binding to purinergic receptors of the P2X family (56, 57). Interestingly, Kolli et al. showed that *L. amazonensis* releases nucleoside diphosphate kinase (NdK), preventing ATP-induced cytolysis of J774 macrophages (58). Further studies are, however, required to access the relevance of NdK in the context of infection. The ERK1/2 pathway also plays a role in the prevention of macrophage apoptosis. Kamir and colleagues described a protein produced by *L. major* that shows structural homology with the human macrophage inhibiting factor (MIF) and exerts similar effects. Indeed this MIF ortholog induced ERK1/2 kinases activation in a CD74-dependent manner, subsequently resulting in the inhibition of macrophage apoptosis *in vitro* (59). The mitochondrial apoptotic pathway is also modulated by *Leishmania*. BMM infected with *L. major* showed enhanced survival that was related with the prevention of cytochrome *c* release by mitochondria (60), observation possibly explained by the involvement of an anti-apoptotic signaling pathway (61). Ruhland and colleagues showed that *L. major* block macrophage apoptosis through the phosphatidylinositol 3′-kinase (PI3K)/protein kinase B (Akt) signaling pathway. Briefly, Akt phosphorylates the pro-apoptotic Bad, deactivating it, and preventing the release of mitochondrial cytochrome *c* (62), which avoids downstream activation of the effector caspase-3 (60). Similar results were obtained with DCs (63, 64). More recently, it was also shown that apoptosis triggered by oxidative burst is prevented by *L. donovani*. Although infected macrophages were capable of ROS production, a complete abrogation of the downstream caspase cascade was observed due to thioredoxin mediated selective induction of suppressors of cytokine signaling (SOCS) proteins (65). A direct responsibility of a parasitic protein was not addressed in these studies, but we cannot exclude the role of phosphoglycans since there are studies that relate them with apoptosis delay in *L. infantum, L. major*, and *L. donovani-*infected macrophages (66, 67). Notwithstanding, the parasites capacity to delay macrophage apoptosis is yet to be shown *in vivo*.

Although parasites delay macrophage death, they cannot prevent it. However, when an infected macrophage dies, *Leishmania* is able to escape. A recent study showed that *L. amazonensis* amastigotes are transferred from cell to cell when the donor host macrophage delivers warning signals of imminent apoptosis (Figure 1). Interestingly, that transfer happens without full exposure of the parasite to the extracellular milieu: the parasites are extruded from the host macrophages within membrane blebs rich in phagolysosomal membrane components, which are in turn phagocytized by nearby macrophages that will then secrete the infection promoting cytokine IL-10 (68).

### Removal of effector T cells by apoptosis

Modulation of cell death is also used by parasites as a way to directly alter the acquired immune response by elimination of effector cells. Felix de Lima et al. showed that apoptosis levels in both peripheral blood and spleen T lymphocytes from *L. infantum* naturally infected dogs are higher in comparison to control animals. The authors concluded that immunosuppression associated with chronic infection is due to accelerated rates of T cell apoptosis, which in turn contributes to white pulp disorganization in the spleen and diminished T cell levels in peripheral blood (69, 70). Furthermore, active human cutaneous leishmaniasis caused by *L. braziliensis* was associated with increased apoptosis of CD8^+^ and CD4^+^ T cells (71). Interestingly, all of these studies linked T cells apoptosis with active disease. However, the mechanisms are yet to be unveiled. The death receptors apoptotic pathway may be involved, as Fas and FasL expression in human splenic lymphocytes is increased in acute disease (72). Furthermore, the correlation between T cell apoptosis and pathophysiological states was further accessed using mouse infection models. In fact infection of susceptible, but not resistant mice with *L. donovani* induced apoptosis of splenic CD4^+^ T cells after *in vitro* stimulation (73). In this case, the mechanisms involved in apoptosis induction, start to be disclosed. Reckling et al. showed that the pro-apoptotic Bcl-2 family member Bim possibly has a role in T cell apoptosis in a mouse model of infection with *L. major* (74). Moreover, in another mouse model infected with *L. donovani*, authors concluded that T cell apoptosis could be related with downregulation of PKC and ERK1/2 activities. Ser/Thr phosphatase seems to have a major role in the initiation of this process by dephosphorylation of key molecules of different T-lymphocyte signaling pathways (75).

Table 1 resumes the topics described above, overviewing the modulation of apoptosis by *Leishmania* in different cell types.

**Table 1:** **Apoptosis modulation during *Leishmania* infection**.

Cell type	Alteration of apoptosis related molecules	Outcome	Key player	References
Neutrophils	Phosphorylation of ERK1/2	Apoptosis inhibition	Unk	(48)
	Upregulation of BX-1 and Bcl-2		
	Inhibition of Bid and pro-caspase 3 processing		
	Prevention of mitochondrial cytochrome *c* release		
	Downregulation of Fas expression		

Macrophages	Decrease of extracellular ATP	Apoptosis inhibition	NdK	(58)
	Phosphorylation of ERK1/2		Lm1740MIF	(59)
	Activation of PI3K/Akt signaling pathway		Unk	(62)
	Deactivation of Bad		
	Induction of SOCS proteins		Thioredoxin	(65)
	Inhibition of pro-caspases 3 and 7 processing		

Dendritic cells	Upregulation of BX-1 and Bcl-2?	Apoptosis inhibition	Unk	(63, 64)
	Prevention of mitochondrial cytochrome *c* release?		
	Inhibition of pro-caspases 3 and 7 processing		

T cells	Upregulation of Bim?	Induction of apoptosis	Unk	(74)
	Deactivation of ERK1/2		Ser/Thre phosphatase	(75)
	Downregulation of Bcl-2		
	Increase of mitochondrial cytochrome *c* release		
	Upregulation of pro-caspase 3 processing		

*Akt, protein kinase B; ERK, extracellular signal-regulated kinases; MIF, macrophage inhibiting factor; NdK, nucleoside diphosphate kinase; SOCS, suppressors of cytokine signaling; Ser/Thr, serine/threonine; Unk, unknown*.

## Overcoming the Immune Leishmanicidal Machinery

*Leishmania* is one of the few intracellular pathogens that can live and replicate inside the harsh environment of a mature phagolysosome. Apart from this parasite, only *Coxiella brunetti* resides during its entire replicative cycle inside that cellular compartment, as reviewed by Voth and Heinzen (76), while other intracellular pathogens that preferentially infect macrophages escape the phagocytic pathway (77). *Leishmania* must, therefore, cope with different effector molecules from the innate immune response in order to survive.

### Avoiding cell lysis and taking advantage of opsonization

The first challenge *Leishmania* encounters in the mammalian host is the complement system (78). Traditionally, promastigote complement resistance is associated with two *Leishmania* glycocalyx components (79): lipophosphoglycan (LPG) and the metalloprotease leishmanolisin (GP63). *Leishmania major* parasites deficient for both these molecules demonstrated high complement sensitivity (80–82). LPG avoids the ultimate step of the complement cascade through prevention of the attachment of the C5b-C9-complex to the parasite surface (83, 84). On the other hand, GP63 inactivates C3b preventing the formation of the C5 convertase complex (85, 86). Albeit, Dominguez et al. showed that under physiological conditions 85–100% of *L. donovani, L. infantum, L. major*, and *L. amazonensis* promastigotes are killed by complement after 2.5 min in human blood (87). Yet, it was also published that as soon as 1 min after *L. amazonensis* and *L. donovani* contact with human blood, infected granulocytes were easily found (88). Therefore, it is essential for the parasite to escape the complement onslaught by quickly entering a phagocytic cell.

Once again *Leishmania* glycocalyx components are used to subvert the innate immune system enhancing the phagocytosis of the parasites. Both GP63 and LPG can directly interact with the host cell surface through binding to the fibronectin receptor and the mannose/fucose receptor, respectively (89–92). Moreover, iC3b, the cleavage product of C3 by GP63, can function as an opsonin (85), and LPG interacts with the early inflammatory C-reactive protein, which triggers phagocytosis (93, 94). Interestingly, iC3b is a ligand of the complement receptor 3 (CR3) (95), and this interaction is directly related with the downregulation of IL-12 production by macrophages (96). The mechanism by which this downregulation happens is not known; however, we may not exclude a toll like receptor (TLR) inhibition since C5a, another complement component, has a negative impact on the TLR-4 induced IL-12 synthesis (97). This may ultimately contribute for the silent entry of the parasites into the host cells.

### Toward a successful differentiation: alterations during the phagolysosome maturation process

After promastigote entry into the host cell, *Leishmania* needs to differentiate to the amastigote form. Since promastigotes cannot survive in the harsh environment of the phagolysosome (low pH, hydrolases), a delay of phagolysosomal fusion was considered essential for the parasite differentiation process (98). Such delay has been described for *L. major, L. infantum*, and *L. donovani* via mechanisms that may or may not involve LPG (98, 99). However, with *L. mexicana* and *L. amazonensis*, this was not proved (100–103). For these parasites, the large parasitophorous vacuoles found in macrophages dilute the hydrolytic enzymes upon lysosome fusion to a level below their effectiveness, allowing promastigotes to differentiate without any requirements of fusion delay (100). In the case of *L. donovani*, it was shown that LPG impairs the association of synaptotagmin V to phagosome membranes, inhibiting the recruitment of the vesicular proton-ATPase and preventing their acidification, allowing promastigote to amastigote differentiation (104). *Leishmania donovani* LPG was also associated with retention of the small GTPase Cdc42 at the phagosome membrane, leading to F-actin accumulation around the phagosome and presumably interfering with vesicle trafficking and phagosome maturation (105, 106).

### Role of GP63 in the defense against antimicrobial peptides

Inside a phagolysosome, fully differentiated or not, *Leishmania* has to deal with other components of the innate immune system: the antimicrobial peptides (AMPs). AMPs are structurally diverse cationic proteins with intrinsic antimicrobial activity, playing normally by disruption of cell surface membranes resulting in osmotic lysis of the pathogen. They can be found both intra and extracellularly, and most of them are constitutively produced and secreted (when applicable) (107, 108). Some human AMPs present activity against *Leishmania*. For example, Kulkarni et al. showed that cathelicidin, an intracellular AMP present in macrophage lysosomes, can kill up to 50% of *L. major* and *L. amazonensis* parasites (109). The same group showed in a different study that α-defensins, produced by neutrophils, also kill *L. major* parasites (110). GP63 play a key role in the defense against these peptides, as it was shown that gp63 KO promastigotes were efficiently killed in a dose dependent manner by AMPs (109).

### Coping with reactive oxygen and nitrogen species (ROS and RNS)

Once inside the host cell, ROS and RNS are the cellular major arms against *Leishmania*. NO^•^ is synthesized by nitric oxide synthase (NOS) during the conversion of l-arginine to l-citrulline, while $O_{2}^{•}$– and other reactive oxygen species (ROS) are generated by the membrane-bound NADPH-dependent oxidases (NOX). These reactive species contribute for the generation of others as ONOO^-^, ${\mathrm{NO}}_{2}^{•}$, and nitrogen trioxide (111). Although NO is considered the most relevant microbicidal molecule, ROS are also associated with disease susceptibility since NOX deficient mice are more susceptible to *L. donovani* and *L. major* infection (112, 113). However, unlike what happens with inducible NOS (iNOS) KO mice, NOX deficient mice eventually control the infection (112–114). Therefore, the parasite needs to somehow neutralize these reactive species and/or prevent their production to avoid a certain death by oxidative stress. The inflammatory cytokine TGF-β produced by infected phagocytes shifts the l-arginine metabolism toward the production of l-ornithine through the activation of arginase (115, 116). This metabolic shift leads to a decrease in NO secretion favoring intracellular *Leishmania* growth (117). Glycocalyx components can also play a role in the protection of *Leishmania* parasites from ROS. A genetic rescue of a *L. amazonensis* GP63 deficient strain increased its intramacrophage survival potential, which was probably related with inhibition of ROS generation (118, 119). In turn, LPG not only prevents ROS generation through inhibition of NOX recruitment to the phagosome membrane, but also directly scavenges these reactive species (81, 120). Glycosylinositolphospholipid (GILP), another component of the glycocalyx, may also be important during the amastigote form, suppressing macrophage iNOS expression and, consequently, NO production (121). Finally, we cannot disregard the intrinsic antioxidant machinery of *Leishmania*, whose most important components are trypanothione synthase and trypanothione reductase. The last one is essential for the fight against ROS and NOS, once disruption of the trypanothione reductase gene renders the parasites susceptible to intracellular killing by macrophages (122). A recent publication shows that *L. donovani* activates multiple own enzymatic mechanisms for the detoxification of ROS and NOS (123). Some of these enzymes have already been associated with protection against reactive species, including the *L. infantum* peroxiredoxins LicTXNPx and LimTXNPx, *L. major* pteridin reductase, and *L. donovani* superoxide dismutase (124–126).

Table 2 discusses the different ways by which components of the *Leishmania* glycocalyx prevents parasite killing by innate immune response.

**Table 2:** **Glycocalyx components: overcoming innate immune leishmanicidal machinery**.

Glycocalyx component	Species	Protective role	Mechanism	References
LPG	*L. major*	Inhibition of complement-mediated lysis	Prevention of attachment of the C5b-C9-complex	(83)
	*L. donovani L. mexicana*	Promotion of phagocytosis to escape the extracellular milieu	Interaction with C-reactive protein and direct binding to phagocytes receptors	(91, 93, 94)
	*L. donovani*	Delay of phagolysosome maturation process	Inhibition of the recruitment of vesicular proton-ATPase	(104)
	*L. donovani*	Reduction of leishmanicidal reactive species	Inhibition of ROS generation	(81, 120)
	*L. major*		ROS scavenging

GP63	*L. major*	Inhibition of complement-mediated lysis	Inactivation of C3b	(85, 86)
	*L. infantum*		
	*L. major L. infantum*	Promotion of phagocytosis to escape the extracellular milieu	The C3b inactivation product functions as an opsonin Direct binding to phagocytes receptors	(85, 89, 92)
	*L. donovani*		
	*L. major*	Prevention of antimicrobial peptide mediated lysis	Proteolytic degradation of the antimicrobial peptides	(109)
	*L. amazonensis*	Reduction of leishmanicidal reactive species	Inhibition of ROS generation	(119)

GILP	*L. major*	Reduction of leishmanicidal reactive species	Suppression of iNOS expression and NO production	(121)

*GILP, glycosylinositolphospholipid; iNOS, inducible nitric oxide synthase; LPG, lipophosphoglycan; NO, nitric oxide; ROS, reactive oxygen species*.

## Modulating the Immune Response through Alteration of Cytokine and Chemokine Signaling and Production

Cytokines are cell signaling mediators, which affect cell function in an autocrine, paracrine, or endocrine manner. Interference with the normal cytokine production is a powerful weapon that the parasite can use for the modulation of immune function. It is generally accepted that production of IL-12 by macrophages and DCs is associated with resistance against *Leishmania*. This cytokine induces naive T cells maturation toward an IFN-γ producing Th1 phenotype (resistant to infection), which in turn induce macrophage M1 activation and elimination of parasites (127, 128). Th2 cytokines, namely IL-4 regarding cutaneous leishmaniasis and IL-10 and TGF-β in the case of visceral disease, have been related with disease susceptibility and progression by induction of an M2 macrophage phenotype (129–131). Therefore, parasites seem to modulate the immune response toward a Th2 phenotype. However, this Th1/Th2 straight polarization seems only to be observed in some murine models, and cannot be fully applicable to human diseases (132). The Th1/Th2 paradigm (reviewed elsewhere) (133, 134) states that Th1 and Th2 cells counter-regulate each other. That would imply that *Leishmania-*induced polarization of the immune response toward a Th2 phenotype would suppress a Th1 immune response. However, what is observed in human disease is a peculiar mixed cytokine response, variable, depending on the infective species (132, 133, 135).

### *Leishmania* modulates TLR signaling

Toll like receptors recognize a variety of pathogen-associated molecular patterns (PAMPs), from proteins to nucleic acids. Upon engagement, TLRs mediate the activation of different transcription factors, such as nuclear factor-κB (NF-κB) and interferon-regulatory factors (IRFs), leading to the production of inflammatory cytokines (136, 137). Induction of cell mediated immunity (138–140) and promotion of NO production (141) are other two known TLR triggered responses against *Leishmania* infection. Nevertheless, the parasite developed strategies that interfere with TLR associated signaling cascades subverting the traditional pro-inflammatory responses. *Ex vivo* experiments suggest that TLR-2 performs a minor role in initiating the synthesis of pro-inflammatory cytokines, namely IL-12, during mice infection with *L. infantum* (142). Chandra et al. showed that *L. donovani* can shift TLR-2 responses toward a Th2 immune response, with downregulation of IL-12 production in macrophages, through MAP kinase inactivation (143). The crosstalk between TLR-2 and CCR-5 (which expression is dependent on the expression of the first one) was also described as relevant in *L. donovani* infection, promoting parasite internalization and inducing a Th2 immune response (144). Moreover, the interaction between TLR2 and LPG was shown do decrease TLR-9 expression leading to a lesser inflammatory profile (145). Nevertheless, the interplay between *Leishmania* and TLRs is highly complex and needs further clarification, once there are several reports showing that LPG-TLR interactions can also result in increase of anti-leishmanial responses by effector cells (146).

The capacity of *Leishmania* to interact with regulatory proteins of the host may also be relevant for TLR signaling modulation. As an example, *L. donovani* exploits a host negative TLR regulator, the deubiquitinating enzyme A20, to inhibit the TLR-2-mediated pro-inflammatory gene expression, consequently suppressing IL-12 and TNF-α production (147). It was also described that *L. donovani*, along with *L. mexicana* and *L. major*, uses the macrophage tyrosine phosphatase SHP-1 to inactivate kinases involved in TLR signaling (148). As happens with TLR-2, *Leishmania* exploits host TLR regulators to deal with TLR-4 activation. Gupta et al. showed that *L. donovani* parasites alter the ubiquitination pattern of TRAF3, preventing its degradation, which is required for the effective cytosolic translocation of the TLR-4-anchored multiprotein complex. As a consequence, NF-κB is silenced leading to a downregulation of IL-12 and TNF-α production (149). Furthermore, *L. amazonensis* amastigotes can suppress TLR-4 activation on DCs via rapid degradation of intracellular signaling proteins (JAK/STAT, NFκB, and IRF) leading to a decrease in IL-12 production (150). The deubiquitinating enzyme A20 also has a role in the inhibition of the TLR-4-mediated pro-inflammatory response. However, in this case, the regulation is an indirect consequence of active disease promoted by the high levels of TGF-β that infected cells produce (151). Another “macrophage imbalance” mediated by TLR-4 signaling manipulation was described by Shweash et al. These authors reported that *L. mexicana* promastigotes are able to prolong and enhance PGE_2_, NO, and arginase production through TLR-4, and consequently achieve the reduction of macrophage released IL-12 (152). Finally, *Leishmania* can impair TLR signaling through prevention of receptor ligand interaction. Here, the player is ectoin-like serine peptidase inhibitor, produced by *L. major*, which inhibits neutrophil elastase and consequently prevents TLR-4 activation (153, 154). Ultimately, TLR-4 signaling inhibition in macrophages induces an M2b phenotype that correlates with higher IL-10 levels and a Th2-type immune response (154). Table 3 collects the data discussed above.

**Table 3:** **Strategies of TLR signaling modulation by *Leishmania*: an overview**.

TLR	Species	Key player	Mechanism of modulation	Reference
TLR 2	*L. donovani*	Unk	Shift to Th2 immune response	(143)
	*L. donovani*	Deubiquitinating enzyme A20	Inhibition of TLR-mediated pro-inflammatory gene expression	(147)
	*L. donovani*		
	*L. mexicana*	SHP-1	Inhibition of TLR-mediated pro-inflammatory gene expression	(148)
	*L. major*		
	*L. major*	LPG	Downregulation of TLR-9 expression	(145)

TLR-4	*L. amazonensis*	Unk	Degradation of intracellular signaling proteins	(150)
	*L. donovani*	Deubiquitinating enzyme A20/SHP-1	Inhibition of TLR-mediated pro-inflammatory gene expression	(151)
	*L. major*	Ecotin-like serine peptidase inhibitor	Shift to Th2 immune response	(154)
	*L. mexicana*	Unk	Enhancement of PGE_2_, NO, and arginase production	(152)

*LPG, lipophosphoglycan; NO, nitric oxide; PGE_2_, prostaglandin E_2_ SHP, sarcoma homology 2 domain phosphatase-1; Th, T helper; TLR, toll like receptor; Unk, unknown*.

### Influencing chemokine production

As an intracellular pathogen, *Leishmania* depends on the initial recruitment of host cells for successful establishment and perpetuation of infection. Chemokines are small proteins that induce and regulate the migration of immune cells, and their expression is known to be modulated by *Leishmania* spp. (41, 155). Several studies reported the upregulation of numerous chemokines (RANTES/CCL5, MIP-1α/CCL3, IP-10/CXCL10, MCP-1/CCL2, MIP-1β/CCL4, MIP-2/CXCL1, and IL-8/CXCL8) after *L. major, L. donovani, L. tropica, L. infantum*, and *L. panamensis* inoculation (156–161). Interestingly, few of these chemokines attract neutrophils, which can be another *Leishmania* mediated immune modulation strategy. Although neutrophils may be a possible vehicle for *Leishmania*, facilitating infection, it was described that exacerbated neutrophil recruitment is associated with parasite killing (162). On the other way, it was also shown that skin lesions of *L. major* infected mice mainly contained Th2 cell-attracting chemokines, such as CCL7 (163, 164). The absence of Th1 cell-attracting chemokines in these lesions may reflect the downregulation of the expression of genes linked with Th1 trafficking, such as the ones coding for CXCR3 chemokines (165). Last but not least, it was described that *Leishmania* may also profit from malnutrition to impair chemokine secretion and to establish infection (158, 166). Interestingly, differential expression of chemokines induced by distinct parasite strains leads to various infection and disease outcomes. As an example, human infection with *L. mexicana* may lead either to a self-healing cutaneous form or to a non-healing cutaneous disease, associated with the increased expression of CCL2 and CCL3, respectively (167). This differential chemokine expression was also seen in human infection with *L. panamensis* (168), and may be related with parasite virulence, once in a mouse model infected with two strains of *L. braziliensis* (highly virulent versus less virulent) a differential chemokine expression profile was observed (169). Elaboration of these studies would be of great interest, particularly regarding the parasite virulence factors responsible for the induction of the chemokine profiles seen in non-healing/severe pathologies, which will unveil new parasite immunomodulatory players.

### Interfering with cytokine production

Although cytokines are important throughout the whole *Leishmania* infectious process, they are fundamental during the acquired immunity phase. IL-12 is mainly produced by APCs, particularly by DCs (170), and is related with important cytokines that mediate very different outcomes of *Leishmania* infection, such as IFN-γ, IL-10, and IL-4. Therefore, the interference with IL-12 is a recurrent phenomenon in *Leishmania* infection. *Leishmania major* was found to deplete cholesterol, inhibiting the assembly of an IL-12-inducing CD40 signalosome and modifying the cell effector functions (171). Others have reported that *L. major* infection directly down-regulates IL-12 production through a CD40 signaling-regulation (172). Furthermore, *L. mexicana* and *L. donovani* were also found to impair LPS-induced IL-12 production by BMM through cysteine proteinase mediated NF-κB degradation (173, 174). Others have correlated IL-12 downregulation with *Leishmania* evasion mechanisms, probably through PI3K/Akt signaling pathway modulation (175–179). In a recent study, *Batf3^-/-^* mice, that lack the major IL-12 producing and cross-presenting subsets CD8α^+^ and CD103^+^ DCs, showed enhanced susceptibility to *L. major* infection partially due to reduced IFN-γ and increased IL-4 and IL-10 secretion (180). IFN-γ is released by Th1 cells triggering the leishmanicidal activity of macrophages via expression of the inducible NO synthase which, in turn, leads to the killing of intracellular *Leishmania* (181). Thus, several reports on prevention of IFN-γ secretion and/or action by the parasite exist. Ray et al. showed that infection of macrophages with *L. donovani* causes a decrease in the phosphorylation of the IFN-γR-α subunit, which consequently affects the receptor expression (182). Furthermore, GP63 was related with reduction of IFN-γ producing cells in BALB/c mice infected with *L. amazonensis* (183). Finally, our group reported that the non-secreted *Leishmania* protein *Lm*S3arp is also associated with downregulation of IFN-γ production by splenocytes (184). It was described that regulatory T cells (Tregs) may have a role in the downregulation of IFN-γ, in a murine model infected with *L. amazonensis* (185). However, it is yet to be unveiled whether and how parasites are able to control these cells. Furthermore, the role of Tregs in infection progression and pathology diverges, depending on the infecting *Leishmania* species. While Tregs are associated with disease exacerbation and parasite persistence, in the infection context with *L. donovani* and *L. major*, respectively, *in vivo* experiments with *L. amazonensis* shown that Tregs aid in disease resolution (185–188). Additionally, Ehrlich et al. demonstrated *in vivo* that both the transfer of Tregs to chronically infected animals with *L. panamensis*, and their treatment with rIL-2/anti-IL-2 Ab complex for Treg expansion contributed for disease amelioration, showing the protective role of Tregs in *L. panamensis* infection and a possible immunotherapeutical role of these cells (189). The immunosuppressive IL-10 has long been associated to visceral disease pathogenesis (190), being not only important in the establishment of infection but also during parasite persistence through the direct inhibition of Th1 cell development, preventing the resolution of the infection (191). In fact, IL-10 receptor blockade or IL-10 KO mice renders animals resistant to *L. donovani* infection (192, 193). The major source of IL-10 in both cutaneous and visceral leishmaniasis is controversial. Some works proposed T regs and Th2 lymphocytes as the main IL-10 producers (190, 194–197), while others claim that Th1 lymphocytes are the main IL-10 source (190, 194, 198–200). Notwithstanding, the parasite can also promote IL-10 production by other cells. For instance, *L. braziliensis* amastigotes and promastigotes induce the secretion of this cytokine by PBMCs (201). This IL-10 secretion was shown to be mediated by phagocytosis of opsonized parasites in an *in vivo* model of low dose infection with *L. major* (202) and also with *L. amazonensis* and *L. mexicana* (203, 204). The *Leishmania* secreted protein *Li*TXN1 is also involved in the promotion of IL-10 production by spenocytes (205). Apart from IL-10, IL-4 also induces Th2 responses (206) and is particularly involved in the promotion of cutaneous leishmaniasis. Tabatabaee et al. suggested that *L. major* secrete immunosuppressive factors that promote IL-4 production by lymphocytes (207). This cytokine was shown to interfere with the synergy of IFN-γ/FasL that contributes to macrophage activation and killing of intracellular *L. major* (208). There is, however, some contradictory studies showing that IL-4 promotes IL-12 production by bone marrow-derived DCs (BMDC) and resistance to the disease (209, 210). Hurdayal et al. clearly showed that DC specific IL-4 receptor alpha (IL-4Rα)-deficient BALB/c mice became hypersusceptible to *L. major* infection, due to a decrease in IL-12 and an increase in IL-10 production by DCs (211). These contradictory observations with IL-4 might be possibly explained by the fact that a low infection dose with *L. major* induces a Th2 response in C57BL/6 mice, whereas high doses induce a Th1 response, both dependent on IL-4 production by lymphocytes (212). Considering the fact that, in average, sandflies transmit not more than 1000 parasites per bite, an induction of Th2 response might be expected in a real situation (213).

Other cytokines have been studied in the context of *Leishmania* infection. IL-17, for instance, has been involved in the outcome of cutaneous leishmaniasis (214–216). Although there are not many studies showing *Leishmania* modulation of this cytokine, some clues exist about how this can happen. Castellano et al. showed that *L. amazonensis* antigens possibly induce a decrease in the percentage of CD3^+^CD4^+^IL-17^+^ human cells, at least in cases of HIV/*Leishmania* co-infection (217). Interestingly, patients with signs of active disease present lower levels of Th17 cytokines (218, 219). Yet, more studies are needed to discover whether *Leishmania* can directly modulate IL-17 production or if it acts on other interlinked cytokines such as IL-6 and IL-23 (201, 216). IL-1β was also shown to influence the clinical course of leishmaniasis, and is strictly related with inflammasome activation, a general but powerful antimicrobial strategy in innate immunity (220). A recent study showed that *Leishmania* can prevent caspase-1-dependent IL-1β activation through a C-type lectin (SIGNR3) mediated signaling process, which consequently favors parasite persistence (221). The parasite key player responsible for this signaling modulation is, however, yet unknown. Finally, IL-13, IL-21, and IL-27 may also have a role in leishmaniasis, either preventing or inducing pathology (222–225).

## Impairing Cellular Function

*Leishmania* is able to control the acquired immunity through the impairment of effector cells function. Antigen processing and presentation by APCs is necessary for the efficient priming of effector T cells which, in turn, will generate a directed and specific immune response (226). Through phagocytosis of parasite debris or intracellular parasite degradation, APCs process and present *Leishmania* antigens (227). Both major histocompatibility complex (MHC) I and MHC II antigen presentation are related with *Leishmania* elimination, although only the second one is essential for complete parasite clearance (212, 228). *Leishmania* can interfere with antigen processing and presentation, consequently modulating once again the immune function.

### *Leishmania* interferes with antigen presentation by professional cells

**Figure 2:**
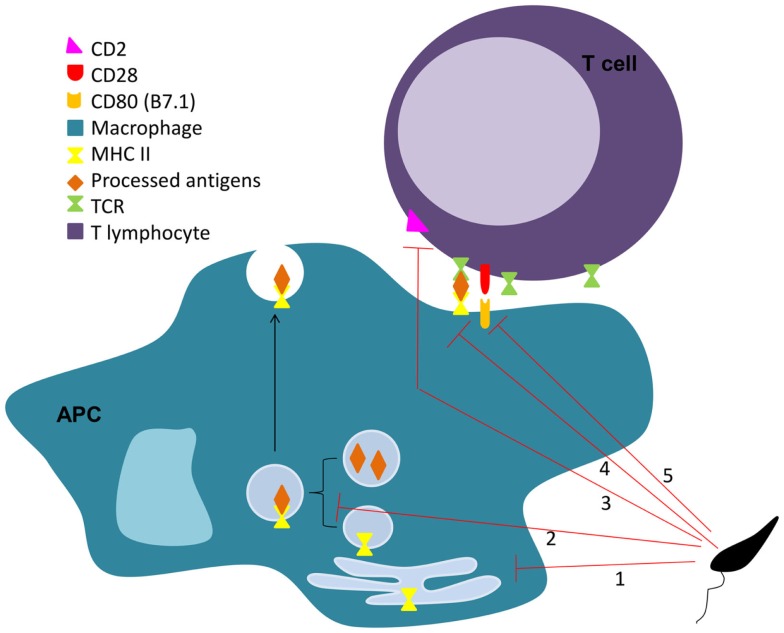
***Leishmania* interferes on MHC II antigen presentation process**. *Leishmania* impairs the antigen presentation process through several mechanisms. The parasite is responsible for the downregulation of MHC II in APC (1), sequestration of antigens from the MHC II pathway (2), limitation of the MHC II-peptide-TCR engagement (3), and down-regulation of co-stimulatory (4), and adhesion molecules (5) on APCs and lymphocytes, respectively.

In 1987, Reiner et al. described that *L. donovani* decreases macrophage expression of both MHC I and MHC II molecules (229). Others have also reported a *L. major* related downregulation of MHC molecules in DCs (230), which can be mediated by direct parasite internalization of these molecules (231–233). Interestingly, *L. donovani* extracellular vesicles were shown to inhibit MHC-II expression in human monocyte-derived DCs (234). Furthermore, both *L. pifanoi* and *L. amazonensis* amastigotes interfere with the macrophage antigen processing process by sequestration of antigens from the MHC II pathway, through a mechanism involving targeted vacuolar fusion (235, 236). However, prevention of surface-expressed MHC class II-peptide complexes is not the only way by which the parasite impairs antigen presentation (Figure 2). *L. donovani* was shown to interfere with BMM antigen presentation by modulating the capacity of surface MHC class II-peptide complexes to engage the T cell receptor (TCR) (237). An increase in the infected cell membrane fluidity by cholesterol depletion and ceramide generation may justify this inefficient engagement (238, 239). Adhesion molecules are also important in the process of antigen presentation. They help during the initiation of contact between APCs and T cells, required for the subsequent formation of the immunological synapse. Bimal et al. reported that particularly CD4^+^, but also CD8^+^ T cells, from patients with active visceral leishmaniasis caused by *L. donovani* express less CD2 than the ones from healthy subjects (240). *In vitro* and *in vivo* studies must, however, be performed to confirm that this downregulation of CD2 in CD4^+^ T cells is caused directly by the parasite. Co-stimulatory molecules are necessary for the full activation of T cells by APCs, which expression can be downregulated by *Leishmania*. For instance, Kaye et al. showed that BMM infected with *L. donovani* expressed lower levels of co-stimulatory molecules B7.1 and heat stable antigen than the non-infected controls (241). Mbow et al. also reported that Langerhan cells of BALB/c mice infected with *L. major* showed a down-regulation of B7.1 expression (242).

### *Leishmania-*induced cellular anergy and exhaustion

The lack of co-stimulatory molecules on APCs, particularly in DCs, can be a consequence of another immune modulation strategy used by *Leishmania*, the inhibition of cell maturation/activation. The induction of cellular unresponsiveness or anergy is the ultimate weapon that *Leishmania* uses in the fight against the immune system. Impairment of APC function was reported by our group. Briefly, BMDC infection with *L. infantum* promastigotes counteracts LPS-triggered activation. Parasites avoided the upregulation of transcription and surface expression of CD40 and CD86 co-stimulatory molecules on BMDC, through activation of the PI3k/Akt pathway and the impairment of NF-κB transcription factor (243). This DCs activation/maturation arrest was also described for *L. amazonensis* infection on mice and human cells (150, 217, 231, 244). *Leishmania* has also been associated with T cell exhaustion (245). Gautam et al. described that IFN-γ production by CD8^+^ effector cells was absent in active human visceral leishmaniasis. These cells expressed elevated levels of Cytotoxic T Lymphocytes Antigen 4 (CTLA-4) and programed death protein 1 (PD1) (246), negative regulators of T cell activation associated with T cell anergy and exhaustion (247). Similar results were also reported by Esch and colleagues, regarding not only CD8, but also CD4 T cells (248). This topic was recently reviewed by our group regarding *Leishmania* and other parasitic infections (249).

## Conclusion

Remarkable progresses were made in the past years in the knowledge of immunomodulation by *Leishmania*. As a result of a long parasite-host co-evolutionary process, this organism can escape or fight the immune system using diverse and complex strategies. However, the knowledge produced is sometimes dispersed and contradictory, reflecting several variables such as infecting species and different infection models. Notwithstanding, it is now clear that the parasite can modulate cell death, alter the maturation process of the phagolysosome, modulate cytokine, and chemokine production by host cells, and impair cell function, in order to silently enter in host cells and successfully differentiate and infect. Furthermore, *Leishmania* released material seems to have by itself some immunomodulatory potential. Therefore, the study of the parasite exoproteome may contribute for the discovery and characterization of the yet unknown arms that the parasite uses to achieve victory against the immune system. The unraveling of the agents responsible for this modulation will help us to define the requirements for infection and disease. This will ultimately become the cornerstone that will contribute to develop novel strategies to fight the disease. Although not discussed in this review, but not less important, the pressure that the parasite exerts in the host cells metabolism is now an area of growing interest. The nascent field of immunometabolism will also contribute significantly for the full understanding of the infectious process.

## Conflict of Interest Statement

The authors declare that the research was conducted in the absence of any commercial or financial relationships that could be construed as a potential conflict of interest.

## References

[1] Bates PA. Transmission of *Leishmania* metacyclic promastigotes by phlebotomine sand flies. Int J Parasitol (2007) 37(10):1097–106. 10.1016/j.ijpara.2007.04.003 17517415 PMC2675784

[2] Beattie L Kaye PM. *Leishmania*-host interactions: what has imaging taught us? Cell Microbiol (2011) 13(11):1659–67. 10.1111/j.1462-5822.2011.01658.x 21819514

[3] Kaye P Scott P. Leishmaniasis: complexity at the host-pathogen interface. Nat Rev Microbiol (2011) 9(8):604–15. 10.1038/nrmicro2608 21747391

[4] WHO. Control of Leishmaniasis: Report of a meeting of the WHO Expert Committee on the Control of Leishmaniasis. WHO Technical Report Series n 949. Switzerland: Published by World Health Organization (2010).

[5] Sinha PK Pandey K Bhattacharya SK. Diagnosis & management of *Leishmania*/HIV co-infection. Indian J Med Res (2005) 121(4):407–14 15817953

[6] Murray HW Berman JD Davies CR Saravia NG. Advances in leishmaniasis. Lancet (2005) 366(9496):1561–77. 10.1016/S0140-6736(05)67629-5 16257344

[7] Desjeux P. Leishmaniasis: current situation and new perspectives. Comp Immunol Microbiol Infect Dis (2004) 27(5):305–18. 10.1016/j.cimid.2004.03.004 15225981

[8] Antinori S Schifanella L Corbellino M. Leishmaniasis: new insights from an old and neglected disease. Eur J Clin Microbiol Infect Dis (2012) 31(2):109–18. 10.1007/s10096-011-1276-0 21533874

[9] Campino LMC. Epidemiologia das leishmanioses em Portugal. Acta Med Port (2010) 23:859–64. 21144327

[10] Salman SM Rubeiz NG Kibbi AG. Cutaneous leishmaniasis: clinical features and diagnosis. Clin Dermatol (1999) 17(3):291–6. 10.1016/S0738-081X(99)00047-4 10384868

[11] Kroidl A Kroidl I Bretzel G Loscher T. Non-healing old world cutaneous leishmaniasis caused by L. infantum in a patient from Spain. BMC Infect Dis (2014) 14:206. 10.1186/1471-2334-14-206 24739742 PMC3991887

[12] Strazzulla A Cocuzza S Pinzone MR Postorino MC Cosentino S Serra A Mucosal leishmaniasis: an underestimated presentation of a neglected disease. Biomed Res Int (2013) 2013:805108. 10.1155/2013/805108 23853773 PMC3703408

[13] Ready PD. Epidemiology of visceral leishmaniasis. Clin Epidemiol (2014) 6:147–54. 10.2147/CLEP.S44267 24833919 PMC4014360

[14] Okwor I Uzonna JE. The immunology of *Leishmania*/HIV co-infection. Immunol Res (2013) 56(1):163–71. 10.1007/s12026-013-8389-8 23504228

[15] Moreno J Alvar J. Canine leishmaniasis: epidemiological risk and the experimental model. Trends Parasitol (2002) 18(9):399–405. 10.1016/S1471-4922(02)02347-4 12377257

[16] Evans KJ Kedzierski L. Development of vaccines against visceral Leishmaniasis. Re Dai Yi Xue Za Zhi (2012) 2012:892817. 10.1155/2012/892817 21912561 PMC3170777

[17] Stockdale L Newton R. A review of preventative methods against human leishmaniasis infection. PLoS Negl Trop Dis (2013) 7(6):e2278. 10.1371/journal.pntd.0002278 23818997 PMC3688540

[18] van Griensven J Balasegaram M Meheus F Alvar J Lynen L Boelaert M. Combination therapy for visceral leishmaniasis. Lancet Infect Dis (2010) 10(3):184–94. 10.1016/S1473-3099(10)70011-6 20185097

[19] Yasinzai M Khan M Nadhman A Shahnaz G. Drug resistance in leishmaniasis: current drug-delivery systems and future perspectives. Fut Med Chem (2013) 5(15):1877–88. 10.4155/fmc.13.143 24144417

[20] Feig C Peter ME. How apoptosis got the immune system in shape. Eur J Immunol (2007) 37(Suppl 1):S61–70. 10.1002/eji.200737462 17972347

[21] Kappler JW Roehm N Marrack P. T cell tolerance by clonal elimination in the thymus. Cell (1987) 49(2):273–80. 10.1016/0092-8674(87)90568-X 3494522

[22] Alderson MR Tough TW Davis-Smith T Braddy S Falk B Schooley KA Fas ligand mediates activation-induced cell death in human T lymphocytes. J Exp Med (1995) 181(1):71–7. 10.1084/jem.181.1.71 7528780 PMC2191813

[23] Dhein J Walczak H Baumler C Debatin KM Krammer PH. Autocrine T-cell suicide mediated by APO-1/(Fas/CD95). Nature (1995) 373(6513):438–41. 10.1038/373438a0 7530335

[24] Mattner J Donhauser N Werner-Felmayer G Bogdan C. NKT cells mediate organ-specific resistance against *Leishmania* major infection. Microbes Infect (2006) 8(2):354–62. 10.1016/j.micinf.2005.07.002 16239118

[25] Kaczanowski S Sajid M Reece SE. Evolution of apoptosis-like programmed cell death in unicellular protozoan parasites. Parasit Vectors (2011) 4:44. 10.1186/1756-3305-4-44 21439063 PMC3077326

[26] Lee N Bertholet S Debrabant A Muller J Duncan R Nakhasi HL. Programmed cell death in the unicellular protozoan parasite *Leishmania*. Cell Death Differ (2002) 9(1):53–64. 10.1038/sj.cdd.4400952 11803374

[27] Proto WR Coombs GH Mottram JC. Cell death in parasitic protozoa: regulated or incidental? Nat Rev Microbiol (2013) 11(1):58–66. 10.1038/nrmicro2929 23202528

[28] van Zandbergen G Bollinger A Wenzel A Kamhawi S Voll R Klinger M *Leishmania* disease development depends on the presence of apoptotic promastigotes in the virulent inoculum. Proc Natl Acad Sci U S A (2006) 103(37):13837–42. 10.1073/pnas.0600843103 16945916 PMC1564231

[29] Wanderley JL Pinto da Silva LH Deolindo P Soong L Borges VM Prates DB Cooperation between apoptotic and viable metacyclics enhances the pathogenesis of Leishmaniasis. PLoS One (2009) 4(5):e5733. 10.1371/journal.pone.0005733 19478944 PMC2684641

[30] Fadok VA Bratton DL Konowal A Freed PW Westcott JY Henson PM. Macrophages that have ingested apoptotic cells in vitro inhibit proinflammatory cytokine production through autocrine/paracrine mechanisms involving TGF-beta, PGE2, and PAF. J Clin Investigat (1998) 101(4):890–8. 10.1172/JCI1112 9466984 PMC508637

[31] Ravichandran KS. Find-me and eat-me signals in apoptotic cell clearance: progress and conundrums. J Exp Med (2010) 207(9):1807–17. 10.1084/jem.20101157 20805564 PMC2931173

[32] Wanderley JL Thorpe PE Barcinski MA Soong L. Phosphatidylserine exposure on the surface of *Leishmania* amazonensis amastigotes modulates in vivo infection and dendritic cell function. Parasite Immunol (2013) 35(3–4):109–19. 10.1111/pim.12019 23163958 PMC3565004

[33] van Zandbergen G Hermann N Laufs H Solbach W Laskay T. *Leishmania* promastigotes release a granulocyte chemotactic factor and induce interleukin-8 release but inhibit gamma interferon-inducible protein 10 production by neutrophil granulocytes. Infect Immun (2002) 70(8):4177–84. 10.1128/IAI.70.8.4177-4184.2002 12117926 PMC128123

[34] Peters NC Egen JG Secundino N Debrabant A Kimblin N Kamhawi S In vivo imaging reveals an essential role for neutrophils in leishmaniasis transmitted by sand flies. Science (2008) 321(5891):970–4. 10.1126/science.1159194 18703742 PMC2606057

[35] Ribeiro-Gomes FL Peters NC Debrabant A Sacks DL. Efficient capture of infected neutrophils by dendritic cells in the skin inhibits the early anti-leishmania response. PLoS Pathog (2012) 8(2):e1002536. 10.1371/journal.ppat.1002536 22359507 PMC3280984

[36] Thalhofer CJ Chen Y Sudan B Love-Homan L Wilson ME. Leukocytes infiltrate the skin and draining lymph nodes in response to the protozoan *Leishmania infantum* chagasi. Infect Immun (2011) 79(1):108–17. 10.1128/IAI.00338-10 20937764 PMC3019875

[37] Beil WJ Meinardus-Hager G Neugebauer DC Sorg C. Differences in the onset of the inflammatory response to cutaneous leishmaniasis in resistant and susceptible mice. J Leukoc Biol (1992) 52(2):135–42. 1506767 10.1002/jlb.52.2.135

[38] Muller K van Zandbergen G Hansen B Laufs H Jahnke N Solbach W Chemokines, natural killer cells and granulocytes in the early course of *Leishmania* major infection in mice. Med Microbiol Immunol (2001) 190(1–2):73–6. 10.1007/s004300100084 11770115

[39] Pompeu ML Freitas LA Santos ML Khouri M Barral-Netto M. Granulocytes in the inflammatory process of BALB/c mice infected by *Leishmania* amazonensis. A quantitative approach. Acta Trop (1991) 48(3):185–93. 10.1016/0001-706X(91)90046-M 1671620

[40] Hassani K Shio MT Martel C Faubert D Olivier M. Absence of metalloprotease GP63 alters the protein content of *Leishmania* exosomes. PLoS One (2014) 9(4):e95007. 10.1371/journal.pone.0095007 24736445 PMC3988155

[41] Matte C Olivier M. *Leishmania*-induced cellular recruitment during the early inflammatory response: modulation of proinflammatory mediators. J Infect Dis (2002) 185(5):673–81. 10.1086/339260 11865425

[42] Wilson ME Innes DJ Sousa AD Pearson RD. Early histopathology of experimental infection with *Leishmania donovani* in hamsters. J Parasitol (1987) 73(1):55–63. 10.2307/3282344 3572666

[43] Geering B Simon HU. Peculiarities of cell death mechanisms in neutrophils. Cell Death Differ (2011) 18(9):1457–69. 10.1038/cdd.2011.75 21637292 PMC3178425

[44] Aga E Katschinski DM van Zandbergen G Laufs H Hansen B Muller K Inhibition of the spontaneous apoptosis of neutrophil granulocytes by the intracellular parasite *Leishmania* major. J Immunol (2002) 169(2):898–905. 10.4049/jimmunol.169.2.898 12097394

[45] Allenbach C Zufferey C Perez C Launois P Mueller C Tacchini-Cottier F. Macrophages induce neutrophil apoptosis through membrane TNF, a process amplified by *Leishmania* major. J Immunol (2006) 176(11):6656–64. 10.4049/jimmunol.176.11.6656 16709824

[46] Santos-Beneit AM Mollinedo F. Expression of genes involved in initiation, regulation, and execution of apoptosis in human neutrophils and during neutrophil differentiation of HL-60 cells. J Leukoc Biol (2000) 67(5):712–24. 10811013 10.1002/jlb.67.5.712

[47] Kilpatrick LE Sun S Mackie D Baik F Li H Korchak HM. Regulation of TNF mediated antiapoptotic signaling in human neutrophils: role of delta-PKC and ERK1/2. J Leukoc Biol (2006) 80(6):1512–21. 10.1189/jlb.0406284 17138860

[48] Sarkar A Aga E Bussmeyer U Bhattacharyya A Moller S Hellberg L Infection of neutrophil granulocytes with *Leishmania* major activates ERK 1/2 and modulates multiple apoptotic pathways to inhibit apoptosis. Med Microbiol Immunol (2013) 202(1):25–35. 10.1007/s00430-012-0246-1 22661217

[49] Menten P Wuyts A Van Damme J. Macrophage inflammatory protein-1. Cytokine Growth Factor Rev (2002) 13(6):455–81. 10.1016/S1359-6101(02)00045-X 12401480

[50] van Zandbergen G Klinger M Mueller A Dannenberg S Gebert A Solbach W Cutting edge: neutrophil granulocyte serves as a vector for *Leishmania* entry into macrophages. J Immunol (2004) 173(11):6521–5. 10.4049/jimmunol.173.11.6521 15557140

[51] Filardy AA Pires DR Nunes MP Takiya CM Freire-de-Lima CG Ribeiro-Gomes FL Proinflammatory clearance of apoptotic neutrophils induces an IL-12(low)IL-10(high) regulatory phenotype in macrophages. J Immunol (2010) 185(4):2044–50. 10.4049/jimmunol.1000017 20660352

[52] John B Hunter CA. Immunology. Neutrophil soldiers or Trojan Horses? Science (2008) 321(5891):917–8. 10.1126/science.1162914 18703727

[53] Afonso L Borges VM Cruz H Ribeiro-Gomes FL DosReis GA Dutra AN Interactions with apoptotic but not with necrotic neutrophils increase parasite burden in human macrophages infected with *Leishmania* amazonensis. J Leukoc Biol (2008) 84(2):389–96. 10.1189/jlb.0108018 18483206

[54] Ritter U Frischknecht F van Zandbergen G. Are neutrophils important host cells for *Leishmania* parasites? Trends Parasitol (2009) 25(11):505–10. 10.1016/j.pt.2009.08.003 19762280

[55] Moore KJ Matlashewski G. Intracellular infection by *Leishmania donovani* inhibits macrophage apoptosis. J Immunol (1994) 152(6):2930–7. 8144893

[56] Ferrari D Los M Bauer MK Vandenabeele P Wesselborg S Schulze-Osthoff K. P2Z purinoreceptor ligation induces activation of caspases with distinct roles in apoptotic and necrotic alterations of cell death. FEBS Lett (1999) 447(1):71–5. 10.1016/S0014-5793(99)00270-7 10218585

[57] Hickman SE el Khoury J Greenberg S Schieren I Silverstein SC. P2Z adenosine triphosphate receptor activity in cultured human monocyte-derived macrophages. Blood (1994) 84(8):2452–6. 7919365

[58] Kolli BK Kostal J Zaborina O Chakrabarty AM Chang KP. *Leishmania*-released nucleoside diphosphate kinase prevents ATP-mediated cytolysis of macrophages. Mol Biochem Parasitol (2008) 158(2):163–75. 10.1016/j.molbiopara.2007.12.010 18242727 PMC2277215

[59] Kamir D Zierow S Leng L Cho Y Diaz Y Griffith J A *Leishmania* ortholog of macrophage migration inhibitory factor modulates host macrophage responses. J Immunol (2008) 180(12):8250–61. 10.4049/jimmunol.180.12.8250 18523291 PMC2668862

[60] Akarid K Arnoult D Micic-Polianski J Sif J Estaquier J Ameisen JC. *Leishmania* major-mediated prevention of programmed cell death induction in infected macrophages is associated with the repression of mitochondrial release of cytochrome c. J Leukoc Biol (2004) 76(1):95–103. 10.1189/jlb.1001877 15075349

[61] Datta SR Dudek H Tao X Masters S Fu H Gotoh Y Akt phosphorylation of BAD couples survival signals to the cell-intrinsic death machinery. Cell (1997) 91(2):231–41. 10.1016/S0092-8674(00)80405-5 9346240

[62] Ruhland A Leal N Kima PE. *Leishmania* promastigotes activate PI3K/Akt signalling to confer host cell resistance to apoptosis. Cell Microbiol (2007) 9(1):84–96. 10.1111/j.1462-5822.2006.00769.x 16889626

[63] Gutierrez-Kobeh L de Oyarzabal E Argueta J Wilkins A Salaiza N Fernandez E Inhibition of dendritic cell apoptosis by *Leishmania mexicana* amastigotes. Parasitol Res (2013) 112(4):1755–62. 10.1007/s00436-013-3334-2 23420408

[64] Valdes-Reyes L Argueta J Moran J Salaiza N Hernandez J Berzunza M *Leishmania mexicana*: inhibition of camptothecin-induced apoptosis of monocyte-derived dendritic cells. Exp Parasitol (2009) 121(3):199–207. 10.1016/j.exppara.2008.10.020 19041644

[65] Srivastav S Basu Ball W Gupta P Giri J Ukil A Das PK. *Leishmania donovani* prevents oxidative burst-mediated apoptosis of host macrophages through selective induction of suppressors of cytokine signaling (SOCS) proteins. J Biol Chem (2014) 289(2):1092–105. 10.1074/jbc.M113.496323 24275663 PMC3887177

[66] Donovan MJ Maciuba BZ Mahan CE McDowell MA. *Leishmania* infection inhibits cycloheximide-induced macrophage apoptosis in a strain-dependent manner. Exp Parasitol (2009) 123(1):58–64. 10.1016/j.exppara.2009.05.012 19500578 PMC2744835

[67] Lisi S Sisto M Acquafredda A Spinelli R Schiavone M Mitolo V Infection with *Leishmania infantum* inhibits actinomycin d-induced apoptosis of human monocytic cell line U-937. J Eukaryot Microbiol (2005) 52(3):211–7. 10.1111/j.1550-7408.2005.00026.x 15926996

[68] Real F Florentino PT Reis LC Ramos-Sanchez EM Veras PS Goto H Cell-to-cell transfer of *Leishmania* amazonensis amastigotes is mediated by immunomodulatory LAMP-rich parasitophorous extrusions. Cell Microbiol (2014). 10.1111/cmi.12311 24824158 PMC4353215

[69] de Lima VM Fattori KR de Souza F Eugenio FR dos Santos PS Rozza DB Apoptosis in T lymphocytes from spleen tissue and peripheral blood of L. (L.) chagasi naturally infected dogs. Vet Parasitol (2012) 184(2–4):147–53. 10.1016/j.vetpar.2011.08.024 21899954

[70] Moreira PR Bandarra Mde B Magalhaes GM Munari DP Machado GF Prandini MM Influence of apoptosis on the cutaneous and peripheral lymph node inflammatory response in dogs with visceral leishmaniasis. Vet Parasitol (2013) 192(1–3):149–57. 10.1016/j.vetpar.2012.09.029 23084537

[71] Bertho AL Santiago MA Da-Cruz AM Coutinho SG. Detection of early apoptosis and cell death in T CD4+ and CD8+ cells from lesions of patients with localized cutaneous leishmaniasis. Braz J Med Biol Res (2000) 33(3):317–25. 10.1590/S0100-879X2000000300010 10719384

[72] Potestio M D’Agostino P Romano GC Milano S Ferlazzo V Aquino A CD4+ CCR5+ and CD4+ CCR3+ lymphocyte subset and monocyte apoptosis in patients with acute visceral leishmaniasis. Immunology (2004) 113(2):260–8. 10.1111/j.1365-2567.2004.01948.x 15379987 PMC1782561

[73] Das G Vohra H Saha B Agrewala JN Mishra GC. *Leishmania donovani* infection of a susceptible host results in apoptosis of Th1-like cells: rescue of anti-leishmanial CMI by providing Th1-specific bystander costimulation. Microbiol Immunol (1998) 42(11):795–801. 10.1111/j.1348-0421.1998.tb02354.x 9886153

[74] Reckling S Divanovic S Karp CL Wojciechowski S Belkaid Y Hildeman D. Proapoptotic Bcl-2 family member Bim promotes persistent infection and limits protective immunity. Infect Immun (2008) 76(3):1179–85. 10.1128/IAI.01093-06 18086806 PMC2258821

[75] Mukherjee P Sen PC Ghose AC. Lymph node cells from BALB/c mice with chronic visceral leishmaniasis exhibiting cellular anergy and apoptosis: involvement of Ser/Thr phosphatase. Apoptosis (2006) 11(11):2013–29. 10.1007/s10495-006-0088-7 17013755

[76] Voth DE Heinzen RA. Lounging in a lysosome: the intracellular lifestyle of Coxiella burnetii. Cell Microbiol (2007) 9(4):829–40. 10.1111/j.1462-5822.2007.00901.x 17381428

[77] Sinai AP Joiner KA. Safe haven: the cell biology of nonfusogenic pathogen vacuoles. Annu Rev Microbiol (1997) 51:415–62. 10.1146/annurev.micro.51.1.415 9343356

[78] Dunkelberger JR Song WC. Complement and its role in innate and adaptive immune responses. Cell Res (2010) 20(1):34–50. 10.1038/cr.2009.139 20010915

[79] Naderer T Vince JE McConville MJ. Surface determinants of *Leishmania* parasites and their role in infectivity in the mammalian host. Curr Mol Med (2004) 4(6):649–65. 10.2174/1566524043360069 15357214

[80] Joshi PB Kelly BL Kamhawi S Sacks DL McMaster WR. Targeted gene deletion in *Leishmania* major identifies leishmanolysin (GP63) as a virulence factor. Mol Biochem Parasitol (2002) 120(1):33–40. 10.1016/S0166-6851(01)00432-7 11849703

[81] Spath GF Garraway LA Turco SJ Beverley SM. The role(s) of lipophosphoglycan (LPG) in the establishment of *Leishmania* major infections in mammalian hosts. Proc Natl Acad Sci U S A (2003) 100(16):9536–41. 10.1073/pnas.1530604100 12869694 PMC170953

[82] Spath GF Lye LF Segawa H Sacks DL Turco SJ Beverley SM. Persistence without pathology in phosphoglycan-deficient *Leishmania* major. Science (2003) 301(5637):1241–3. 10.1126/science.1087499 12947201

[83] Puentes SM Da Silva RP Sacks DL Hammer CH Joiner KA. Serum resistance of metacyclic stage *Leishmania* major promastigotes is due to release of C5b-9. J Immunol (1990) 145(12):4311–6. 2147941

[84] McConville MJ Turco SJ Ferguson MA Sacks DL. Developmental modification of lipophosphoglycan during the differentiation of *Leishmania* major promastigotes to an infectious stage. EMBO J (1992) 11(10):3593–600. 1396559 10.1002/j.1460-2075.1992.tb05443.x PMC556818

[85] Brittingham A Morrison CJ McMaster WR McGwire BS Chang KP Mosser DM. Role of the *Leishmania* surface protease gp63 in complement fixation, cell adhesion, and resistance to complement-mediated lysis. J Immunol (1995) 155(6):3102–11. 7673725

[86] Yao C Gaur Dixit U Barker JH Teesch LM Love-Homan L Donelson JE Attenuation of *Leishmania infantum* chagasi metacyclic promastigotes by sterol depletion. Infect Immun (2013) 81(7):2507–17. 10.1128/IAI.00214-13 23630964 PMC3697599

[87] Dominguez M Moreno I Lopez-Trascasa M Torano A. Complement interaction with trypanosomatid promastigotes in normal human serum. J Exp Med (2002) 195(4):451–9. 10.1084/jem.20011319 11854358 PMC2193616

[88] Moreno I Dominguez M Cabanes D Aizpurua C Torano A. Kinetic analysis of ex vivo human blood infection by *Leishmania*. PLoS Negl Trop Dis (2010) 4(7):e743. 10.1371/journal.pntd.0000743 20644618 PMC2903471

[89] Rizvi FS Ouaissi MA Marty B Santoro F Capron A. The major surface protein of *Leishmania* promastigotes is a fibronectin-like molecule. Eur J Immunol (1988) 18(3):473–6. 10.1002/eji.1830180323 2965651

[90] Blackwell JM Ezekowitz RA Roberts MB Channon JY Sim RB Gordon S. Macrophage complement and lectin-like receptors bind *Leishmania* in the absence of serum. J Exp Med (1985) 162(1):324–31. 10.1084/jem.162.1.324 3891904 PMC2187694

[91] Wilson ME Pearson RD. Evidence that *Leishmania donovani* utilizes a mannose receptor on human mononuclear phagocytes to establish intracellular parasitism. J Immunol (1986) 136(12):4681–8. 3711662

[92] Brittingham A Chen G McGwire BS Chang KP Mosser DM. Interaction of *Leishmania* gp63 with cellular receptors for fibronectin. Infect Immun (1999) 67(9):4477–84. 10456889 10.1128/iai.67.9.4477-4484.1999 PMC96767

[93] Culley FJ Harris RA Kaye PM McAdam KP Raynes JG. C-reactive protein binds to a novel ligand on *Leishmania donovani* and increases uptake into human macrophages. J Immunol (1996) 156(12):4691–6. 8648114

[94] Talamas-Rohana P Wright SD Lennartz MR Russell DG. Lipophosphoglycan from *Leishmania mexicana* promastigotes binds to members of the CR3, p150,95 and LFA-1 family of leukocyte integrins. J Immunol (1990) 144(12):4817–24. 1972169

[95] Ueno N Bratt CL Rodriguez NE Wilson ME. Differences in human macrophage receptor usage, lysosomal fusion kinetics and survival between logarithmic and metacyclic *Leishmania infantum* chagasi promastigotes. Cell Microbiol (2009) 11(12):1827–41. 10.1111/j.1462-5822.2009.01374.x 19702651 PMC2788030

[96] Marth T Kelsall BL. Regulation of interleukin-12 by complement receptor 3 signaling. J Exp Med (1997) 185(11):1987–95. 10.1084/jem.185.11.1987 9166428 PMC2196332

[97] Hawlisch H Belkaid Y Baelder R Hildeman D Gerard C Kohl J. C5a negatively regulates toll-like receptor 4-induced immune responses. Immunity (2005) 22(4):415–26. 10.1016/j.immuni.2005.02.006 15845447

[98] Desjardins M Descoteaux A. Inhibition of phagolysosomal biogenesis by the *Leishmania* lipophosphoglycan. J Exp Med (1997) 185(12):2061–8. 10.1084/jem.185.12.2061 9182677 PMC2196352

[99] Rodriguez NE Gaur U Wilson ME. Role of caveolae in *Leishmania chagasi* phagocytosis and intracellular survival in macrophages. Cell Microbiol (2006) 8(7):1106–20. 10.1111/j.1462-5822.2006.00695.x 16819964

[100] Duclos S Desjardins M. Subversion of a young phagosome: the survival strategies of intracellular pathogens. Cell Microbiol (2000) 2(5):365–77. 10.1046/j.1462-5822.2000.00066.x 11207592

[101] Forestier CL Machu C Loussert C Pescher P Spath GF. Imaging host cell-*Leishmania* interaction dynamics implicates parasite motility, lysosome recruitment, and host cell wounding in the infection process. Cell Host Microbe (2011) 9(4):319–30. 10.1016/j.chom.2011.03.011 21501831

[102] Real F Pouchelet M Rabinovitch M. *Leishmania* (L.) amazonensis: fusion between parasitophorous vacuoles in infected bone-marrow derived mouse macrophages. Exp Parasitol (2008) 119(1):15–23. 10.1016/j.exppara.2007.12.013 18346736

[103] Russell DG Xu S Chakraborty P. Intracellular trafficking and the parasitophorous vacuole of *Leishmania mexicana*-infected macrophages. J Cell Sci (1992) 103(Pt 4):1193–210. 1487496 10.1242/jcs.103.4.1193

[104] Vinet AF Fukuda M Turco SJ Descoteaux A. The *Leishmania donovani* lipophosphoglycan excludes the vesicular proton-ATPase from phagosomes by impairing the recruitment of synaptotagmin V. PLoS Pathog (2009) 5(10):e1000628. 10.1371/journal.ppat.1000628 19834555 PMC2757729

[105] Holm A Tejle K Magnusson KE Descoteaux A Rasmusson B. *Leishmania donovani* lipophosphoglycan causes periphagosomal actin accumulation: correlation with impaired translocation of PKCalpha and defective phagosome maturation. Cell Microbiol (2001) 3(7):439–47. 10.1046/j.1462-5822.2001.00127.x 11437830

[106] Lodge R Descoteaux A. *Leishmania donovani* promastigotes induce periphagosomal F-actin accumulation through retention of the GTPase Cdc42. Cell Microbiol (2005) 7(11):1647–58. 10.1111/j.1462-5822.2005.00582.x 16207251

[107] Ganz T Lehrer RI. Antibiotic peptides from higher eukaryotes: biology and applications. Mol Med Today (1999) 5(7):292–7. 10.1016/S1357-4310(99)01490-2 10377520

[108] Guani-Guerra E Santos-Mendoza T Lugo-Reyes SO Teran LM. Antimicrobial peptides: general overview and clinical implications in human health and disease. Clin Immunol (2010) 135(1):1–11. 10.1016/j.clim.2009.12.004 20116332

[109] Kulkarni MM McMaster WR Kamysz E Kamysz W Engman DM McGwire BS. The major surface-metalloprotease of the parasitic protozoan, *Leishmania*, protects against antimicrobial peptide-induced apoptotic killing. Mol Microbiol (2006) 62(5):1484–97. 10.1111/j.1365-2958.2006.05459.x 17074074

[110] Kulkarni MM McMaster WR Kamysz W McGwire BS. Antimicrobial peptide-induced apoptotic death of *Leishmania* results from calcium-de pend ent, caspase-independent mitochondrial toxicity. J Biol Chem (2009) 284(23):15496–504. 10.1074/jbc.M809079200 19357081 PMC2708846

[111] Gostner JM Becker K Fuchs D Sucher R. Redox regulation of the immune response. Redox Rep (2013) 18(3):88–94. 10.1179/1351000213Y.0000000044 23601165 PMC6837572

[112] Murray HW Nathan CF. Macrophage microbicidal mechanisms in vivo: reactive nitrogen versus oxygen intermediates in the killing of intracellular visceral *Leishmania donovani*. J Exp Med (1999) 189(4):741–6. 10.1084/jem.189.4.741 9989990 PMC2192937

[113] Blos M Schleicher U Soares Rocha FJ Meissner U Rollinghoff M Bogdan C. Organ-specific and stage-dependent control of *Leishmania* major infection by inducible nitric oxide synthase and phagocyte NADPH oxidase. Eur J Immunol (2003) 33(5):1224–34. 10.1002/eji.200323825 12731047

[114] Wei XQ Charles IG Smith A Ure J Feng GJ Huang FP Altered immune responses in mice lacking inducible nitric oxide synthase. Nature (1995) 375(6530):408–11. 10.1038/375408a0 7539113

[115] Modolell M Corraliza IM Link F Soler G Eichmann K. Reciprocal regulation of the nitric oxide synthase/arginase balance in mouse bone marrow-derived macrophages by TH1 and TH2 cytokines. Eur J Immunol (1995) 25(4):1101–4. 10.1002/eji.1830250436 7537672

[116] Boutard V Havouis R Fouqueray B Philippe C Moulinoux JP Baud L. Transforming growth factor-beta stimulates arginase activity in macrophages. Implications for the regulation of macrophage cytotoxicity. J Immunol (1995) 155(4):2077–84. 7636258

[117] Iniesta V Gomez-Nieto LC Molano I Mohedano A Carcelen J Miron C Arginase I induction in macrophages, triggered by Th2-type cytokines, supports the growth of intracellular *Leishmania* parasites. Parasite Immunol (2002) 24(3):113–8. 10.1046/j.1365-3024.2002.00444.x 11982856

[118] Buchmuller-Rouiller Y Mauel J. Correlation between enhanced oxidative metabolism and leishmanicidal activity in activated macrophages from healer and nonhealer mouse strains. J Immunol (1986) 136(10):3884–90. 3009612

[119] McGwire B Chang KP. Genetic rescue of surface metalloproteinase (gp63)-deficiency in *Leishmania* amazonensis variants increases their infection of macrophages at the early phase. Mol Biochem Parasitol (1994) 66(2):345–7. 10.1016/0166-6851(94)90160-0 7808483

[120] Lodge R Diallo TO Descoteaux A. *Leishmania donovani* lipophosphoglycan blocks NADPH oxidase assembly at the phagosome membrane. Cell Microbiol (2006) 8(12):1922–31. 10.1111/j.1462-5822.2006.00758.x 16848789

[121] Proudfoot L Nikolaev AV Feng GJ Wei WQ Ferguson MA Brimacombe JS Regulation of the expression of nitric oxide synthase and leishmanicidal activity by glycoconjugates of *Leishmania* lipophosphoglycan in murine macrophages. Proc Natl Acad Sci U S A (1996) 93(20):10984–9. 10.1073/pnas.93.20.10984 8855295 PMC38270

[122] Tovar J Cunningham ML Smith AC Croft SL Fairlamb AH. Down- regulation of *Leishmania donovani* trypanothione reductase by heterologous expression of a trans-dominant mutant homologue: effect on parasite intracellular survival. Proc Natl Acad Sci U S A (1998) 95(9):5311–6. 10.1073/pnas.95.9.5311 9560272 PMC20257

[123] Sardar AH Kumar S Kumar A Purkait B Das S Sen A Proteome changes associated with *Leishmania donovani* promastigote adaptation to oxidative and nitrosative stresses. J Proteomics (2013) 81:185–99. 10.1016/j.jprot.2013.01.011 23376486

[124] Nare B Garraway LA Vickers TJ Beverley SM. PTR1-dependent synthesis of tetrahydrobiopterin contributes to oxidant susceptibility in the trypanosomatid protozoan parasite *Leishmania* major. Curr Genet (2009) 55(3):287–99. 10.1007/s00294-009-0244-z 19396443 PMC2759280

[125] Ghosh S Goswami S Adhya S. Role of superoxide dismutase in survival of *Leishmania* within the macrophage. Biochem J (2003) 369(Pt 3):447–52. 10.1042/BJ20021684 12459037 PMC1223130

[126] Castro H Sousa C Santos M Cordeiro-da-Silva A Flohe L Tomas AM. Complementary antioxidant defense by cytoplasmic and mitochondrial peroxiredoxins in *Leishmania infantum*. Free Radic Biol Med (2002) 33(11):1552–62. 10.1016/S0891-5849(02)01089-4 12446213

[127] Diaz-Gandarilla JA Osorio-Trujillo C Hernandez-Ramirez VI Talamas-Rohana P. PPAR activation induces M1 macrophage polarization via cPLA(2)-COX-2 inhibition, activating ROS production against *Leishmania mexicana*. Biomed Res Int (2013) 2013:215283. 10.1155/2013/215283 23555077 PMC3600276

[128] Guler R Afshar M Arendse B Parihar SP Revaz-Breton M Leitges M PKCdelta regulates IL-12p40/p70 production by macrophages and dendritic cells, driving a type 1 healer phenotype in cutaneous leishmaniasis. Eur J Immunol (2011) 41(3):706–15. 10.1002/eji.201040985 21287553

[129] Mia S Warnecke A Zhang XM Malmstrom V Harris RA. An optimized protocol for human M2 macrophages using M-CSF and IL-4/IL-10/TGF-beta yields a dominant immunosuppressive phenotype. Scand J Immunol (2014) 79(5):305–14. 10.1111/sji.12162 24521472 PMC4282403

[130] Satoskar A Bluethmann H Alexander J. Disruption of the murine interleukin-4 gene inhibits disease progression during *Leishmania mexicana* infection but does not increase control of *Leishmania donovani* infection. Infect Immun (1995) 63(12):4894–9. 7591152 10.1128/iai.63.12.4894-4899.1995 PMC173701

[131] Gautam S Kumar R Maurya R Nylen S Ansari N Rai M IL-10 neutralization promotes parasite clearance in splenic aspirate cells from patients with visceral leishmaniasis. J Infect Dis (2011) 204(7):1134–7. 10.1093/infdis/jir461 21881130 PMC3164427

[132] Nylen S Gautam S. Immunological perspectives of leishmaniasis. J Global Infect Dis (2010) 2(2):135–46. 10.4103/0974-777X.62876 PMC2889653 20606969

[133] McMahon-Pratt D Alexander J. Does the *Leishmania* major paradigm of pathogenesis and protection hold for New World cutaneous leishmaniases or the visceral disease? Immunol Rev (2004) 201:206–24. 10.1111/j.0105-2896.2004.00190.x 15361243

[134] Muraille E Leo O. Revisiting the Th1/Th2 paradigm. Scand J Immunol (1998) 47(1):1–9. 10.1111/j.1365-3083.1998-47-1.00383.x 9467651

[135] Ahmed S Colmenares M Soong L Goldsmith-Pestana K Munstermann L Molina R Intradermal infection model for pathogenesis and vaccine studies of murine visceral leishmaniasis. Infect Immun (2003) 71(1):401–10. 10.1128/IAI.71.1.401-410.2003 12496190 PMC143149

[136] Akira S Uematsu S Takeuchi O. Pathogen recognition and innate immunity. Cell (2006) 124(4):783–801. 10.1016/j.cell.2006.02.015 16497588

[137] Broz P Monack DM. Newly described pattern recognition receptors team up against intracellular pathogens. Nat Rev Immunol (2013) 13(8):551–65. 10.1038/nri3479 23846113

[138] Liese J Schleicher U Bogdan C. TLR9 signaling is essential for the innate NK cell response in murine cutaneous leishmaniasis. Eur J Immunol (2007) 37(12):3424–34. 10.1002/eji.200737182 18034422

[139] Schleicher U Liese J Knippertz I Kurzmann C Hesse A Heit A NK cell activation in visceral leishmaniasis requires TLR9, myeloid DCs, and IL-12, but is independent of plasmacytoid DCs. J Exp Med (2007) 204(4):893–906. 10.1084/jem.20061293 17389237 PMC2118560

[140] Karmakar S Bhaumik SK Paul J De T. TLR4 and NKT cell synergy in immunotherapy against visceral leishmaniasis. PLoS Pathog (2012) 8(4):e1002646. 10.1371/journal.ppat.1002646 22511870 PMC3325212

[141] Paul J Karmakar S De T. TLR-mediated distinct IFN-gamma/IL-10 pattern induces protective immunity against murine visceral leishmaniasis. Eur J Immunol (2012) 42(8):2087–99. 10.1002/eji.201242428 22622993

[142] Silvestre R Silva AM Cordeiro-da-Silva A Ouaissi A. The contribution of Toll-like receptor 2 to the innate recognition of a *Leishmania infantum* silent information regulator 2 protein. Immunology (2009) 128(4):484–99. 10.1111/j.1365-2567.2009.03132.x 19930041 PMC2792133

[143] Chandra D Naik S. *Leishmania donovani* infection down-regulates TLR2-stimulated IL-12p40 and activates IL-10 in cells of macrophage/monocytic lineage by modulating MAPK pathways through a contact-dependent mechanism. Clin Exp Immunol (2008) 154(2):224–34. 10.1111/j.1365-2249.2008.03741.x 18778366 PMC2612710

[144] Majumdar SB Bhattacharya P Bhattacharjee S Majumder S Banerjee S Majumdar S. Toll like receptor 2 and CC chemokine receptor 5 cluster in the lipid raft enhances the susceptibility of *Leishmania donovani* infection in macrophages. Indian J Exp Biol (2014) 52(1):17–29. 24617012

[145] Srivastava S Pandey SP Jha MK Chandel HS Saha B. *Leishmania* expressed lipophosphoglycan interacts with Toll-like receptor (TLR)-2 to decrease TLR-9 expression and reduce anti-leishmanial responses. Clin Exp Immunol (2013) 172(3):403–9. 10.1111/cei.12074 23600828 PMC3646439

[146] Faria MS Reis FC Lima AP. Toll-like receptors in *Leishmania* infections: guardians or promoters? J Parasitol Res (2012) 2012:930257. 10.1155/2012/930257 22523644 PMC3317170

[147] Srivastav S Kar S Chande AG Mukhopadhyaya R Das PK. *Leishmania donovani* exploits host deubiquitinating enzyme A20, a negative regulator of TLR signaling, to subvert host immune response. J Immunol (2012) 189(2):924–34. 10.4049/jimmunol.1102845 22685311

[148] Abu-Dayyeh I Shio MT Sato S Akira S Cousineau B Olivier M. Leishmania-induced IRAK-1 inactivation is mediated by SHP-1 interacting with an evolutionarily conserved KTIM motif. PLoS Negl Trop Dis (2008) 2(12):e305. 10.1371/journal.pntd.0000305 19104650 PMC2596967

[149] Gupta P Giri J Srivastav S Chande AG Mukhopadhyaya R Das PK *Leishmania donovani* targets tumor necrosis factor receptor-associated factor (TRAF) 3 for impairing TLR4-mediated host response. FASEB J (2014) 28(4):1756–68. 10.1096/fj.13-238428 24391131

[150] Xin L Li K Soong L. Down-regulation of dendritic cell signaling pathways by *Leishmania* amazonensis amastigotes. Mol Immunol (2008) 45(12):3371–82. 10.1016/j.molimm.2008.04.018 18538399 PMC2583126

[151] Das S Pandey K Kumar A Sardar AH Purkait B Kumar M TGF-beta1 re-programs TLR4 signaling in *L. donovani* infection: enhancement of SHP-1 and ubiquitin-editing enzyme A20. Immunol Cell Biol (2012) 90(6):640–54. 10.1038/icb.2011.80 21968712

[152] Shweash M Adrienne McGachy H Schroeder J Neamatallah T Bryant CE Millington O *Leishmania mexicana* promastigotes inhibit macrophage IL-12 production via TLR-4 dependent COX-2, iNOS and arginase-1 expression. Mol Immunol (2011) 48(15–16):1800–8. 10.1016/j.molimm.2011.05.013 21664694 PMC3173610

[153] Faria MS Calegari-Silva TC de Carvalho Vivarini A Mottram JC Lopes UG Lima AP. Role of protein kinase R in the killing of *Leishmania* major by macrophages in response to neutrophil elastase and TLR4 via TNFalpha and IFNbeta. FASEB J (2014) 28:3050–63. 10.1096/fj.13-245126 24732131 PMC4210457

[154] Faria MS Reis FC Azevedo-Pereira RL Morrison LS Mottram JC Lima AP. *Leishmania* inhibitor of serine peptidase 2 prevents TLR4 activation by neutrophil elastase promoting parasite survival in murine macrophages. J Immunol (2011) 186(1):411–22. 10.4049/jimmunol.1002175 21098233 PMC3119636

[155] Teixeira MJ Teixeira CR Andrade BB Barral-Netto M Barral A. Chemokines in host-parasite interactions in leishmaniasis. Trends Parasitol (2006) 22(1):32–40. 10.1016/j.pt.2005.11.010 16310413

[156] Antoniazi S Price HP Kropf P Freudenberg MA Galanos C Smith DF Chemokine gene expression in toll-like receptor-competent and -deficient mice infected with *Leishmania* major. Infect Immun (2004) 72(9):5168–74. 10.1128/IAI.72.9.5168-5174.2004 15322011 PMC517484

[157] Forget G Matte C Siminovitch KA Rivest S Pouliot P Olivier M. Regulation of the *Leishmania*-induced innate inflammatory response by the protein tyrosine phosphatase SHP-1. Eur J Immunol (2005) 35(6):1906–17. 10.1002/eji.200526037 15902687

[158] Kumar V Bimal S Singh SK Chaudhary R Das S Lal C *Leishmania donovani*: dynamics of *L. donovani* evasion of innate immune cell attack due to malnutrition in visceral leishmaniasis. Nutrition (2014) 30(4):449–58. 10.1016/j.nut.2013.10.003 24607302

[159] Navas A Vargas DA Freudzon M McMahon-Pratt D Gore Saravia N Gomez MA. Chronicity of dermal leishmaniasis caused by *Leishmania* panamensis is associated with parasite mediated induction of chemokine gene expression. Infect Immun (2014) 82(7):2872–80. 10.1128/IAI.01133-13 24752514 PMC4097649

[160] Kobets T Havelkova H Grekov I Volkova V Vojtiskova J Slapnickova M Genetics of host response to *Leishmania* tropica in mice – different control of skin pathology, chemokine reaction, and invasion into spleen and liver. PLoS Negl Trop Dis (2012) 6(6):e1667. 10.1371/journal.pntd.0001667 22679519 PMC3367980

[161] Menezes-Souza D Guerra-Sa R Carneiro CM Vitoriano-Souza J Giunchetti RC Teixeira-Carvalho A Higher expression of CCL2, CCL4, CCL5, CCL21, and CXCL8 chemokines in the skin associated with parasite density in canine visceral leishmaniasis. PLoS Negl Trop Dis (2012) 6(4):e1566. 10.1371/journal.pntd.0001566 22506080 PMC3323520

[162] Forget G Siminovitch KA Brochu S Rivest S Radzioch D Olivier M. Role of host phosphotyrosine phosphatase SHP-1 in the development of murine leishmaniasis. Eur J Immunol (2001) 31(11):3185–96. 10.1002/1521-4141(200111)31:11<3185::AID-IMMU3185>3.0.CO;2-J 11745335

[163] Katzman SD Fowell DJ. Pathogen-imposed skewing of mouse chemokine and cytokine expression at the infected tissue site. J Clin Invest (2008) 118(2):801–11. 10.1172/JCI33174 18188454 PMC2176190

[164] Roebrock K Sunderkotter C Munck NA Wolf M Nippe N Barczyk K Epidermal expression of I-TAC (Cxcl11) instructs adaptive Th2-type immunity. FASEB J (2014) 28(4):1724–34. 10.1096/fj.13-233593 24398292

[165] Lazarski CA Ford J Katzman SD Rosenberg AF Fowell DJ. IL-4 attenuates Th1-associated chemokine expression and Th1 trafficking to inflamed tissues and limits pathogen clearance. PLoS One (2013) 8(8):e71949. 10.1371/journal.pone.0071949 23991011 PMC3753298

[166] Ibrahim MK Barnes JL Osorio EY Anstead GM Jimenez F Osterholzer JJ Deficiency of lymph node resident dendritic cells and dysregulation of DC chemoattractants in a malnourished mouse model of *Leishmania donovani* infection. Infect Immun (2014) 82(8):3098–112. 10.1128/IAI.01778-14 24818662 PMC4136237

[167] Ritter U Moll H Laskay T Brocker E Velazco O Becker I Differential expression of chemokines in patients with localized and diffuse cutaneous American leishmaniasis. J Infect Dis (1996) 173(3):699–709. 10.1093/infdis/173.3.699 8627035

[168] Navas A Vargas DA Freudzon M McMahon-Pratt D Saravia NG Gomez MA. Chronicity of dermal Leishmaniasis caused by *Leishmania* panamensis is associated with parasite-mediated induction of chemokine gene expression. Infect Immun (2014) 82(7):2872–80. 10.1128/IAI.01133-13 24752514 PMC4097649

[169] Teixeira MJ Fernandes JD Teixeira CR Andrade BB Pompeu ML Santana da Silva J Distinct *Leishmania braziliensis* isolates induce different paces of chemokine expression patterns. Infect Immun (2005) 73(2):1191–5. 10.1128/IAI.73.2.1191-1195.2005 15664963 PMC546968

[170] Gorak PM Engwerda CR Kaye PM. Dendritic cells, but not macrophages, produce IL-12 immediately following *Leishmania donovani* infection. Eur J Immunol (1998) 28(2):687–95. 10.1002/(SICI)1521-4141(199802)28:02<687::AID-IMMU687>3.0.CO;2-N 9521079

[171] Rub A Dey R Jadhav M Kamat R Chakkaramakkil S Majumdar S Cholesterol depletion associated with *Leishmania* major infection alters macrophage CD40 signalosome composition and effector function. Nat Immunol (2009) 10(3):273–80. 10.1038/ni.1705 19198591

[172] Srivastava N Sudan R Saha B. CD40-modulated dual-specificity phosphatases MAPK phosphatase (MKP)-1 and MKP-3 reciprocally regulate *Leishmania* major infection. J Immunol (2011) 186(10):5863–72. 10.4049/jimmunol.1003957 21471446

[173] Cameron P McGachy A Anderson M Paul A Coombs GH Mottram JC Inhibition of lipopolysaccharide-induced macrophage IL-12 production by *Leishmania mexicana* amastigotes: the role of cysteine peptidases and the NF-kappaB signaling pathway. J Immunol (2004) 173(5):3297–304. 10.4049/jimmunol.173.5.3297 15322192

[174] Carrera L Gazzinelli RT Badolato R Hieny S Muller W Kuhn R *Leishmania* promastigotes selectively inhibit interleukin 12 induction in bone marrow-derived macrophages from susceptible and resistant mice. J Exp Med (1996) 183(2):515–26. 10.1084/jem.183.2.515 8627163 PMC2192469

[175] Belkaid Y Butcher B Sacks DL. Analysis of cytokine production by inflammatory mouse macrophages at the single-cell level: selective impairment of IL-12 induction in *Leishmania*-infected cells. Eur J Immunol (1998) 28(4):1389–400. 10.1002/(SICI)1521-4141(199804)28:04<1389::AID-IMMU1389>3.0.CO;2-1 9565379

[176] Kim S Elkon KB Ma X. Transcriptional suppression of interleukin-12 gene expression following phagocytosis of apoptotic cells. Immunity (2004) 21(5):643–53. 10.1016/j.immuni.2004.09.009 15539151

[177] Ruhland A Kima PE. Activation of PI3K/Akt signaling has a dominant negative effect on IL-12 production by macrophages infected with *Leishmania* amazonensis promastigotes. Exp Parasitol (2009) 122(1):28–36. 10.1016/j.exppara.2008.12.010 19186178 PMC2669696

[178] Stuart LM Lucas M Simpson C Lamb J Savill J Lacy-Hulbert A. Inhibitory effects of apoptotic cell ingestion upon endotoxin-driven myeloid dendritic cell maturation. J Immunol (2002) 168(4):1627–35. 10.4049/jimmunol.168.4.1627 11823490

[179] Sutterwala FS Noel GJ Clynes R Mosser DM. Selective suppression of interleukin-12 induction after macrophage receptor ligation. J Exp Med (1997) 185(11):1977–85. 10.1084/jem.185.11.1977 9166427 PMC2196339

[180] Ashok D Schuster S Ronet C Rosa M Mack V Lavanchy C Cross-presenting dendritic cells are required for control of *Leishmania* major infection. Eur J Immunol (2014) 44(5):1422–32. 10.1002/eji.201344242 24643576

[181] Wang ZE Reiner SL Zheng S Dalton DK Locksley RM. CD4+ effector cells default to the Th2 pathway in interferon gamma-deficient mice infected with *Leishmania* major. J Exp Med (1994) 179(4):1367–71. 10.1084/jem.179.4.1367 7908325 PMC2191434

[182] Ray M Gam AA Boykins RA Kenney RT. Inhibition of interferon-gamma signaling by *Leishmania donovani*. J Infect Dis (2000) 181(3):1121–8. 10.1086/315330 10720539

[183] Thiakaki M Kolli B Chang KP Soteriadou K. Down-regulation of gp63 level in *Leishmania* amazonensis promastigotes reduces their infectivity in BALB/c mice. Microbes Infect (2006) 8(6):1455–63. 10.1016/j.micinf.2006.01.006 16698300

[184] Cordeiro-Da-Silva A Borges MC Guilvard E Ouaissi A. Dual role of the *Leishmania* major ribosomal protein S3a homologue in regulation of T- and B-cell activation. Infect Immun (2001) 69(11):6588–96. 10.1128/IAI.69.11.6588-6596.2001 11598026 PMC100031

[185] Ji J Masterson J Sun J Soong L. CD4+CD25+ regulatory T cells restrain pathogenic responses during *Leishmania* amazonensis infection. J Immunol (2005) 174(11):7147–53. 10.4049/jimmunol.174.11.7147 15905558 PMC2812412

[186] Gupta G Majumdar S Adhikari A Bhattacharya P Mukherjee AK Majumdar SB Treatment with IP-10 induces host-protective immune response by regulating the T regulatory cell functioning in *Leishmania donovani*-infected mice. Med Microbiol Immunol (2011) 200(4):241–53. 10.1007/s00430-011-0197-y 21533785

[187] Martin S Agarwal R Murugaiyan G Saha B. CD40 expression levels modulate regulatory T cells in *Leishmania donovani* infection. J Immunol (2010) 185(1):551–9. 10.4049/jimmunol.0902206 20525887

[188] Mendez S Reckling SK Piccirillo CA Sacks D Belkaid Y. Role for CD4(+) CD25(+) regulatory T cells in reactivation of persistent leishmaniasis and control of concomitant immunity. J Exp Med (2004) 200(2):201–10. 10.1084/jem.20040298 15263027 PMC2212012

[189] Ehrlich A Moreno Castilho T Goldsmith-Pestana K Chae WJ Bothwell AL Sparwasser T The Immunotherapeutic Role of Regulatory T Cells in *Leishmania* (Viannia) panamensis Infection. J Immunol (2014) 193(6):2961–70. 10.4049/jimmunol.1400728 25098291 PMC4170189

[190] Nylen S Maurya R Eidsmo L Manandhar KD Sundar S Sacks D. Splenic accumulation of IL-10 mRNA in T cells distinct from CD4+CD25+ (Foxp3) regulatory T cells in human visceral leishmaniasis. J Exp Med (2007) 204(4):805–17. 10.1084/jem.20061141 17389235 PMC2118563

[191] Okwor I Uzonna J. Persistent parasites and immunologic memory in cutaneous leishmaniasis: implications for vaccine designs and vaccination strategies. Immunol Res (2008) 41(2):123–36. 10.1007/s12026-008-8016-2 18389179

[192] Murray HW Lu CM Mauze S Freeman S Moreira AL Kaplan G Interleukin-10 (IL-10) in experimental visceral leishmaniasis and IL-10 receptor blockade as immunotherapy. Infect Immun (2002) 70(11):6284–93. 10.1128/IAI.70.11.6284-6293.2002 12379707 PMC130311

[193] Murphy ML Wille U Villegas EN Hunter CA Farrell JP. IL-10 mediates susceptibility to *Leishmania donovani* infection. Eur J Immunol (2001) 31(10):2848–56. 10.1002/1521-4141(2001010)31:10<2848::AID-IMMU2848>3.3.CO;2-K 11592059

[194] Anderson CF Oukka M Kuchroo VJ Sacks D. CD4(+)CD25(-)Foxp3(-) Th1 cells are the source of IL-10-mediated immune suppression in chronic cutaneous leishmaniasis. J Exp Med (2007) 204(2):285–97. 10.1084/jem.20061886 17283207 PMC2118728

[195] Belkaid Y Piccirillo CA Mendez S Shevach EM Sacks DL. CD4+CD25+ regulatory T cells control *Leishmania* major persistence and immunity. Nature (2002) 420(6915):502–7. 10.1038/nature01152 12466842

[196] Brelaz MC de Oliveira AP de Almeida AF de Assis Souza M Medeiros AC de Brito ME Antigenic fractions of *Leishmania* (Viannia) braziliensis: the immune response characterization of patients at the initial phase of disease. Parasite Immunol (2012) 34(4):236–9. 10.1111/j.1365-3024.2012.01351.x 22394223

[197] Saha S Mondal S Ravindran R Bhowmick S Modak D Mallick S IL-10- and TGF-beta-mediated susceptibility in kala-azar and post-kala-azar dermal leishmaniasis: the significance of amphotericin B in the control of *Leishmania donovani* infection in India. J Immunol (2007) 179(8):5592–603. 10.4049/jimmunol.179.8.5592 17911647

[198] Owens BM Beattie L Moore JW Brown N Mann JL Dalton JE IL-10-producing Th1 cells and disease progression are regulated by distinct CD11c(+) cell populations during visceral leishmaniasis. PLoS Pathog (2012) 8(7):e1002827. 10.1371/journal.ppat.1002827 22911108 PMC3406093

[199] Pagan AJ Peters NC Debrabant A Ribeiro-Gomes F Pepper M Karp CL Tracking antigen-specific CD4+ T cells throughout the course of chronic *Leishmania* major infection in resistant mice. Eur J Immunol (2013) 43(2):427–38. 10.1002/eji.201242715 23109292 PMC4086308

[200] Resende M Moreira D Augusto J Cunha J Neves B Cruz MT Leishmania-infected MHC class IIhigh dendritic cells polarize CD4+ T cells toward a nonprotective T-bet+ IFN-gamma+ IL-10+ phenotype. J Immunol (2013) 191(1):262–73. 10.4049/jimmunol.1203518 23729437

[201] Gomes CM Avila LR Pinto SA Duarte FB Pereira LI Abrahamsohn IA *Leishmania braziliensis* amastigotes stimulate production of IL-1beta, IL-6, IL-10 and TGF-beta by peripheral blood mononuclear cells from nonendemic area healthy residents. Parasite Immunol (2014) 36(5):225–31. 10.1111/pim.12109 24575815

[202] Padigel UM Farrell JP. Control of infection with *Leishmania* major in susceptible BALB/c mice lacking the common gamma-chain for FcR is associated with reduced production of IL-10 and TGF-beta by parasitized cells. J Immunol (2005) 174(10):6340–5. 10.4049/jimmunol.174.10.6340 15879134

[203] Yang Z Mosser DM Zhang X. Activation of the MAPK, ERK, following *Leishmania* amazonensis infection of macrophages. J Immunol (2007) 178(2):1077–85. 10.4049/jimmunol.178.2.1077 17202371 PMC2643020

[204] Buxbaum LU. *Leishmania mexicana* infection induces IgG to parasite surface glycoinositol phospholipids that can induce IL-10 in mice and humans. PLoS Negl Trop Dis (2013) 7(5):e2224. 10.1371/journal.pntd.0002224 23675550 PMC3649955

[205] Santarem N Silvestre R Tavares J Silva M Cabral S Maciel J Immune response regulation by *Leishmania* secreted and nonsecreted antigens. J Biomed Biotechnol (2007) 2007(6):85154. 10.1155/2007/85154 17710243 PMC1940321

[206] Himmelrich H Launois P Maillard I Biedermann T Tacchini-Cottier F Locksley RM In BALB/c mice, IL-4 production during the initial phase of infection with *Leishmania* major is necessary and sufficient to instruct Th2 cell development resulting in progressive disease. J Immunol (2000) 164(9):4819–25. 10.4049/jimmunol.164.9.4819 10779790

[207] Tabatabaee PA Abolhassani M Mahdavi M Nahrevanian H Azadmanesh K. *Leishmania* major: secreted antigens of *Leishmania* major promastigotes shift the immune response of the C57BL/6 mice toward Th2 in vitro. Exp Parasitol (2011) 127(1):46–51. 10.1016/j.exppara.2010.06.033 20603118

[208] Chakour R Allenbach C Desgranges F Charmoy M Mauel J Garcia I A new function of the Fas-FasL pathway in macrophage activation. J Leukoc Biol (2009) 86(1):81–90. 10.1189/jlb.1008590 19380712

[209] Hochrein H O’Keeffe M Luft T Vandenabeele S Grumont RJ Maraskovsky E Interleukin (IL)-4 is a major regulatory cytokine governing bioactive IL-12 production by mouse and human dendritic cells. J Exp Med (2000) 192(6):823–33. 10.1084/jem.192.6.823 10993913 PMC2193283

[210] Biedermann T Zimmermann S Himmelrich H Gumy A Egeter O Sakrauski AK IL-4 instructs TH1 responses and resistance to *Leishmania* major in susceptible BALB/c mice. Nat Immunol (2001) 2(11):1054–60. 10.1038/ni725 11600887

[211] Hurdayal R Nieuwenhuizen NE Revaz-Breton M Smith L Hoving JC Parihar SP Deletion of IL-4 receptor alpha on dendritic cells renders BALB/c mice hypersusceptible to *Leishmania* major infection. PLoS Pathog (2013) 9(10):e1003699. 10.1371/journal.ppat.1003699 24204259 PMC3812013

[212] Uzonna JE Joyce KL Scott P. Low dose *Leishmania* major promotes a transient T helper cell type 2 response that is down-regulated by interferon gamma-producing CD8+ T cells. J Exp Med (2004) 199(11):1559–66. 10.1084/jem.20040172 15184505 PMC2211781

[213] Maia C Seblova V Sadlova J Votypka J Volf P. Experimental transmission of *Leishmania infantum* by two major vectors: a comparison between a viscerotropic and a dermotropic strain. PLoS Negl Trop Dis (2011) 5(6):e1181. 10.1371/journal.pntd.0001181 21695108 PMC3114756

[214] Bacellar O Faria D Nascimento M Cardoso TM Gollob KJ Dutra WO Interleukin 17 production among patients with American cutaneous leishmaniasis. J Infect Dis (2009) 200(1):75–8. 10.1086/599380 19476435 PMC2732405

[215] Katara GK Ansari NA Singh A Ramesh V Salotra P. Evidence for involvement of Th17 type responses in post kala azar dermal leishmaniasis (PKDL). PLoS Negl Trop Dis (2012) 6(6):e1703. 10.1371/journal.pntd.0001703 22724038 PMC3378621

[216] Soong L Henard CA Melby PC. Immunopathogenesis of non-healing American cutaneous leishmaniasis and progressive visceral leishmaniasis. Semin Immunopathol (2012) 34(6):735–51. 10.1007/s00281-012-0350-8 23053396 PMC4111229

[217] Castellano LR Llaguno M Silva MV Machado JR Correia D Silva-Vergara ML Immunophenotyping of circulating T cells in a mucosal leishmaniasis patient coinfected with HIV. Rev Soc Bras Med Trop (2011) 44(4):520–1. 10.1590/S0037-86822011000400025 21860904

[218] Ghosh K Sharma G Saha A Kar S Das PK Ukil A. Successful therapy of visceral leishmaniasis with curdlan involves T-helper 17 cytokines. J Infect Dis (2013) 207(6):1016–25. 10.1093/infdis/jis771 23255562

[219] Pitta MG Romano A Cabantous S Henri S Hammad A Kouriba B IL-17 and IL-22 are associated with protection against human kala azar caused by *Leishmania donovani*. J Clin Invest (2009) 119(8):2379–87. 10.1172/JCI38813 19620772 PMC2719936

[220] Hise AG Tomalka J Ganesan S Patel K Hall BA Brown GD An essential role for the NLRP3 inflammasome in host defense against the human fungal pathogen Candida albicans. Cell Host Microbe (2009) 5(5):487–97. 10.1016/j.chom.2009.05.002 19454352 PMC2824856

[221] Lefevre L Lugo-Villarino G Meunier E Valentin A Olagnier D Authier H The C-type lectin receptors dectin-1, MR, and SIGNR3 contribute both positively and negatively to the macrophage response to *Leishmania infantum*. Immunity (2013) 38(5):1038–49. 10.1016/j.immuni.2013.04.010 23684988

[222] Ansari NA Kumar R Gautam S Nylen S Singh OP Sundar S IL-27 and IL-21 are associated with T cell IL-10 responses in human visceral leishmaniasis. J Immunol (2011) 186(7):3977–85. 10.4049/jimmunol.1003588 21357266 PMC3076633

[223] Santangeli L McCluney NA Hathorn I Shakeel M Anderson C. Leishmaniasis presenting to the otolaryngologist: a rare but important cause of persistent hoarseness. J Laryngol Otol (2009) 123(10):1181–3. 10.1017/S0022215109004216 19128519

[224] Nigg AP Zahn S Ruckerl D Holscher C Yoshimoto T Ehrchen JM Dendritic cell-derived IL-12p40 homodimer contributes to susceptibility in cutaneous leishmaniasis in BALB/c mice. J Immunol (2007) 178(11):7251–8. 10.4049/jimmunol.178.11.7251 17513774

[225] Hurdayal R Brombacher F. The role of IL-4 and IL-13 in cutaneous Leishmaniasis. Immunol Lett (2014) 161(2):179–83. 10.1016/j.imlet.2013.12.022 24412597

[226] Kaye PM. Costimulation and the regulation of antimicrobial immunity. Immunol Today (1995) 16(9):423–7. 10.1016/0167-5699(95)80018-2 7546205

[227] Overath P Aebischer T. Antigen presentation by macrophages harboring intravesicular pathogens. Parasitol Today (1999) 15(8):325–32. 10.1016/S0169-4758(99)01473-8 10407380

[228] Locksley RM Reiner SL Hatam F Littman DR Killeen N. Helper T cells without CD4: control of leishmaniasis in CD4-deficient mice. Science (1993) 261(5127):1448–51. 10.1126/science.8367726 8367726

[229] Reiner NE Ng W McMaster WR. Parasite-accessory cell interactions in murine leishmaniasis. II. *Leishmania donovani* suppresses macrophage expression of class I and class II major histocompatibility complex gene products. J Immunol (1987) 138(6):1926–32. 2434567

[230] Muraille E De Trez C Pajak B Torrentera FA De Baetselier P Leo O Amastigote load and cell surface phenotype of infected cells from lesions and lymph nodes of susceptible and resistant mice infected with *Leishmania* major. Infect Immun (2003) 71(5):2704–15. 10.1128/IAI.71.5.2704-2715.2003 12704145 PMC153240

[231] Prina E Abdi SZ Lebastard M Perret E Winter N Antoine JC. Dendritic cells as host cells for the promastigote and amastigote stages of *Leishmania* amazonensis: the role of opsonins in parasite uptake and dendritic cell maturation. J Cell Sci (2004) 117(Pt 2):315–25. 10.1242/jcs.00860 14657281

[232] Bennett CL Colledge L Richards HE Reay PA Blackburn CC Aebischer T. Uncompromised generation of a specific H-2DM-dependent peptide-MHC class II complex from exogenous antigen in *Leishmania mexicana*-infected dendritic cells. Eur J Immunol (2003) 33(12):3504–13. 10.1002/eji.200323425 14635061

[233] Antoine JC Lang T Prina E Courret N Hellio R. H-2M molecules, like MHC class II molecules, are targeted to parasitophorous vacuoles of *Leishmania*-infected macrophages and internalized by amastigotes of *L. amazonensis* and *L. mexicana*. J Cell Sci (1999) 112(Pt 15):2559–70. 10393812 10.1242/jcs.112.15.2559

[234] Silverman JM Clos J Horakova E Wang AY Wiesgigl M Kelly I *Leishmania* exosomes modulate innate and adaptive immune responses through effects on monocytes and dendritic cells. J Immunol (2010) 185(9):5011–22. 10.4049/jimmunol.1000541 20881185

[235] Kima PE Soong L Chicharro C Ruddle NH McMahon-Pratt D. *Leishmania*-infected macrophages sequester endogenously synthesized parasite antigens from presentation to CD4+ T cells. Eur J Immunol (1996) 26(12):3163–9. 10.1002/eji.1830261249 8977318

[236] Prina E Jouanne C de Souza Lao S Szabo A Guillet JG Antoine JC. Antigen presentation capacity of murine macrophages infected with *Leishmania* amazonensis amastigotes. J Immunol (1993) 151(4):2050–61. 8102156

[237] Meier CL Svensson M Kaye PM. *Leishmania*-induced inhibition of macrophage antigen presentation analyzed at the single-cell level. J Immunol (2003) 171(12):6706–13. 10.4049/jimmunol.171.12.6706 14662874

[238] Chakraborty D Banerjee S Sen A Banerjee KK Das P Roy S. *Leishmania donovani* affects antigen presentation of macrophage by disrupting lipid rafts. J Immunol (2005) 175(5):3214–24. 10.4049/jimmunol.175.5.3214 16116212

[239] Majumder S Dey R Bhattacharjee S Rub A Gupta G Bhattacharyya Majumdar S *Leishmania*-induced biphasic ceramide generation in macrophages is crucial for uptake and survival of the parasite. J Infect Dis (2012) 205(10):1607–16. 10.1093/infdis/jis229 22517914

[240] Bimal S Singh SK Sinha S Pandey K Sinha PK Ranjan A *Leishmania donovani*: role of CD2 on CD4+ T-cell function in Visceral Leishmaniasis. Exp Parasitol (2008) 118(2):238–46. 10.1016/j.exppara.2007.08.009 17904553

[241] Kaye PM Rogers NJ Curry AJ Scott JC. Deficient expression of co-stimulatory molecules on *Leishmania*-infected macrophages. Eur J Immunol (1994) 24(11):2850–4. 10.1002/eji.1830241140 7525308

[242] Mbow ML DeKrey GK Titus RG. *Leishmania* major induces differential expression of costimulatory molecules on mouse epidermal cells. Eur J Immunol (2001) 31(5):1400–9. 10.1002/1521-4141(200105)31:5<1400::AID-IMMU1400>3.0.CO;2-J 11466703

[243] Neves BM Silvestre R Resende M Ouaissi A Cunha J Tavares J Activation of phosphatidylinositol 3-kinase/Akt and impairment of nuclear factor-kappaB: molecular mechanisms behind the arrested maturation/activation state of *Leishmania infantum*-infected dendritic cells. Am J Pathol (2010) 177(6):2898–911. 10.2353/ajpath.2010.100367 21037075 PMC2993270

[244] Favali C Tavares N Clarencio J Barral A Barral-Netto M Brodskyn C. *Leishmania* amazonensis infection impairs differentiation and function of human dendritic cells. J Leukoc Biol (2007) 82(6):1401–6. 10.1189/jlb.0307187 17890507

[245] Mueller DL. Mechanisms maintaining peripheral tolerance. Nat Immunol (2010) 11(1):21–7. 10.1038/ni.1817 20016506

[246] Gautam S Kumar R Singh N Singh AK Rai M Sacks D CD8 T cell exhaustion in human visceral leishmaniasis. J Infect Dis (2014) 209(2):290–9. 10.1093/infdis/jit401 23922369 PMC3873784

[247] Chen L. Co-inhibitory molecules of the B7-CD28 family in the control of T-cell immunity. Nat Rev Immunol (2004) 4(5):336–47. 10.1038/nri1349 15122199

[248] Esch KJ Juelsgaard R Martinez PA Jones DE Petersen CA. Programmed death 1-mediated T cell exhaustion during visceral leishmaniasis impairs phagocyte function. J Immunol (2013) 191(11):5542–50. 10.4049/jimmunol.1301810 24154626 PMC3896087

[249] Rodrigues V Cordeiro-da-Silva A Laforge M Ouaissi A Akharid K Silvestre R Impairment of T cell function in parasitic infections. PLoS Negl Trop Dis (2014) 8(2):e2567. 10.1371/journal.pntd.0002567 24551250 PMC3923671

